# An enactivist-inspired mathematical model of cognition

**DOI:** 10.3389/fnbot.2022.846982

**Published:** 2022-09-30

**Authors:** Vadim Weinstein, Basak Sakcak, Steven M. LaValle

**Affiliations:** Center for Ubiquitous Computing, Faculty of Information Technology and Electrical Engineering, University of Oulu, Oulu, Finland

**Keywords:** enactivism, transition systems, automaton, cognitive modeling, information spaces, robotics

## Abstract

In this paper we start from the philosophical position in cognitive science known as enactivism. We formulate five basic enactivist tenets that we have carefully identified in the relevant literature as the main underlying principles of that philosophy. We then develop a mathematical framework to talk about cognitive systems (both artificial and natural) which complies with these enactivist tenets. In particular we pay attention that our mathematical modeling does not attribute contentful symbolic representations to the agents, and that the agent's nervous system or brain, body and environment are modeled in a way that makes them an inseparable part of a greater totality. The long-term purpose for which this article sets the stage is to create a mathematical foundation for cognition which is in line with enactivism. We see two main benefits of doing so: (1) It enables enactivist ideas to be more accessible for computer scientists, AI researchers, roboticists, cognitive scientists, and psychologists, and (2) it gives the philosophers a mathematical tool which can be used to clarify their notions and help with their debates. Our main notion is that of a sensorimotor system which is a special case of a well studied notion of a transition system. We also consider related notions such as labeled transition systems and deterministic automata. We analyze a notion called *sufficiency* and show that it is a very good candidate for a foundational notion in the “mathematics of cognition from an enactivist perspective.” We demonstrate its importance by proving a uniqueness theorem about the minimal sufficient refinements (which correspond in some sense to an optimal attunement of an organism to its environment) and by showing that sufficiency corresponds to known notions such as sufficient history information spaces. In the end, we tie it all back to the enactivist tenets.

## 1. Introduction: Mathematizing enactivism

The premise of this paper is to lay down a logical framework for analyzing agency in a novel way, inspired by enactivism. Classically, mathematical and logical models of cognition are in line with the cognitivist paradigm in that they rely on the notion of symbolic representation and do not emphasize embodiment or enactment (Newell and Simon, 1972; Fodor, 2008; Gallistel and King, 2009; Rescorla, 2016). Cognitivism presumes that the world possesses objective structure and the contentful information of this structure is acquired and represented by the cognitive agent. This aligns well with the classical model-theoretic paradigm. In this paradigm a formal language is describing a static model (such as when sentences in the language of rings describe algebraic structures—such as rings).

In the cognitivist analogy, the agent possesses (“in its head”) formulas of the language and the model is the world or the environment of the agent. If the formulas possessed by the agent hold in the model, then the agent's representation of the world is correct; otherwise, it is incorrect. Such view of cognitive agency is rejected by the enactivists either weakly or strongly depending on the branch of enactivism. For example, radical enactivism (Hutto and Myin, 2012, 2017) rejects this view strongly. Our question for this paper is: What would the mathematical logic of cognition look like, if even the radical enactivists were to accept it?

We do not take part in the cognitivist-enactivist, or the representationalist-antirepresentationalist debate (Pezzulo et al., 2011; O'Regan and Block, 2012; Gallagher, 2018; Fuchs, 2020). Rather, we take a somewhat extreme enactivist and antirepresentational view as our axiomatic starting point and as a theoretical explanatory target. Then we develop a mathematical theory that attempts to account for cognition in a way congruent with this view. Even though most forms of enactivism (even radical ones) have room for representation, it is not our main goal at the moment to bridge the gap between “basic minds” and “scaffolded minds,” to use terminology of (Hutto and Myin, 2017). Thus, in this terminology, we are going to explore a mathematical (only) of *basic minds*.

**Figure 1:**
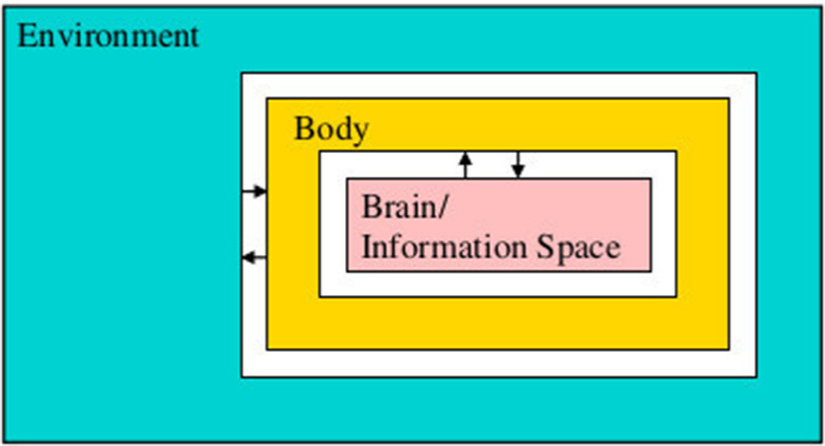
The environment, body, and nervous system (or brain) will be modeled as inseparable parts of a coupled transition system.

The following “axioms” we take as fundamentals for our work:

(EA1) Embodiment. “From a third-person perspective the organism-environment is taken as the explanatory unit” (Gallagher, 2017). The environment, the body, and the nervous system are inseparable parts of the system which they form by coupling; see Figure 1. They cannot be meaningfully understood in isolation from each other. “Mentality is in all cases concretely constituted by, and thus literally consists of, the extensive ways in which organisms interact with their environments, where the relevant ways of interacting involve, but are not exclusively restricted to, what goes on in brains” (Embodiment Thesis Hutto and Myin, 2012).
(EA2) Groundedness. The brain does not “acquire” or “possess” contentful states, representations, or manipulate semantic information in any other way. “Mentality-constituting interactions are grounded in, shaped by, and explained by nothing more, or other, than the history of an organism's previous interactions. Nothing other than its history of active engaging structures or explains an organism's current interactive tendencies.” [Developmental-Explanatory Thesis (Hutto and Myin, 2012)].
(EA3) Emergence. The crucial properties of the brain-body-environment system from the point of view of cognition emerge from the embodiment, the brain-body-environment coupling, the situatedness, and the skills of the agent. The agent's and the environment's prior structure come together to facilitate new structure which emerges through the sensorimotor engagement. “[T]he mind and world arise together in enaction, [but] their manner of arising is not arbitrary” (i.e. it is structured) (Varela et al., 1992).
(EA4) Attunement. Agents differ in their ways of attunement and adaptation to their environments, and in the skills they have. A *skill* is a potential possibility to engage *reliably* in complex sensorimotor interactions with the environment (Gallagher, 2017).
(EA5) Perception. Sensing and perceiving are not the same thing. Perception arises from skillful sensorimotor activity. To perceive is to become better attuned to the environment. O'Regan and Noë (2001) and Noë (2004) “Perception and action, sensorium and motorium, are linked together as successively emergent and mutually selecting patterns.” Varela et al. (1992).

The mathematics we use to capture those ideas is a mixture of known and new concepts from theoretical robotics, (non-)deterministic automata and transition systems theory, and dynamical systems (Goranko and Otto, 2007). It will also build upon the *information spaces* framework, introduced in LaValle (2006) as a unified way to model sensing, actuation, and planning in robotics; the framework itself builds upon earlier ideas such as dynamic games with imperfect information (von Neumann and Morgenstern, 1944; Başar and Olsder, 1995), control with imperfect state information (Kumar and Varaiya, 1986; Bertsekas, 2001), knowledge states (Lozano-Pérez et al., 1984; Erdmann, 1993), perceptual equivalence classes (Donald and Jennings, 1991; Donald, 1995), maze and graph-exploring automata (Shannon, 1952; Blum and Kozen, 1978; Fraigniaud et al., 2005), and belief spaces (Kaelbling et al., 1998; Roy and Gordon, 2003).

Although information spaces refer to “information,” they are not directly related to Shannon's *information theory* (Shannon, 1948), which came later than von Neumann's use of information in the context of sequential game theory. Neither does “information” here refer to content-bearing information. One important intuition behind the information in information spaces is that more information corresponds to narrowing down the space of possibilities (for example of future sensorimotor interactions).

The main mathematical concept of this paper is a *sensorimotor system* (SM-system), which is a special case of a transition system. Sensorimotor systems can describe the body-brain system, the body-environment system as well as other parts of the brain-body-environment system. Given two SM-systems they can be *coupled* to produce another (third) SM-system. Mathematically, the coupling operation is akin to a direct product. We introduce several notions that describe the coupling of the agent and the environment from an outside perspective (not from the perspective of the agent or the environment). The main notion is that of *sufficiency*. In some sense it guarantees that the coupling is of “high fidelity.” It does not compare “internal” models of the agent to “external” states of affairs. Rather it asks whether the way in which the agent engages in sensorimotor patterns is well structured. The notion of *sufficiency* compares the sensorimiotor capacity of the agent *to itself* by asking whether the past sensorimotor patterns (in a given environment) determine reliably the future sensorimotor patterns. We then introduce several related notions. The *degree of insufficiency* is a measure by which various agents can be compared in their coupling versatility (Def 4.11). *Minimal sufficient refinement* is a concept that can be used in the most vivid ways to illustrate how the sensorimotor interaction “enacts” properties of the brain-body-environment system. The notion of minimal sufficient refinement ties together mathematics of sensorimotor systems and the philosophical ideas of emergence, structural coupling and enactment of the “world we inhabit” (cf. Varela et al., 1992); see Example 4.25. We prove the uniqueness of minimal sufficient refinements (Theorem 4.19) and point out their connection to the notions of bisimulation and sufficient information mappings. *Strategic sufficiency* is a mathematically more challenging concept, but has appealing properties in the philosophical and practical sense. A sensor mapping is strategically sufficient *for* some subset of the state space *G*, if that sensor can (in principle) be used by the agent to reach *G* ^1^. Again, any sensor mapping has minimal strategic refinements, but this time they are not unique. Different minimal refinements in this case can be thought of as different adaptations to the same environmental demands.

Mathematically, sufficiency is a relative concept to some known notions in theoretical computer science and robotics: that of *bisimulation* in automata and Kripke model theory (Goranko and Otto, 2007), and *sufficient information mappings* in information spaces theory (LaValle, 2006).

Minimal sufficient refinements lead to unique classifications of agent-environment states that “emerge” from the way in which the agent is coupled to the environment, not merely from the way the environment is structured on its own. Thus, the world is simultaneously objectively existing (from the global “god” perspective), but also “brought about” by the agent.

This should be enough to answer the two questions that, according to Paolo (2018), any embodied theory of cognition should be able to provide precise answers to: What is its conception of bodies? What central role do bodies play in this theory different from the roles they play in traditional computationalism?

Section 2 introduces the basics of transition and SM-systems, their coupling, and other mathematical constructs such as quotients. Section 3 illustrates the introduced notions with detailed examples. Section 4 introduces the notion of sufficiency, sufficient refinements, and minimal sufficient refinements. We will prove the uniqueness theorem for the latter and illustrate the notions in a computational setting. We will explore the importance of sufficiency and related notions for the enactivist way of looking at cognitive organization. Finally, Section 5 ties the mathematics back to the philosophical premises.

## 2. Transition systems and sensorimotor systems

At the most abstract level, the central concept for our mathematical theory is that of a *transition system*. This is a standard definition from automata theory (for instance Goranko and Otto, 2007):

**Definition 2.1.** A *transition system* is a triple (*X, U, T*) where *X* is the *state space* (mathematically it is just a set), *U* is the set of names for outgoing transitions (another set), and *T* ⊆ *X* × *U* × *X* is a ternary relation.

The intuitive interpretation of (*X, U, T*) is that it is possible to transition from the state *x*_1_ ∈ *X* to the state *x*_2_ ∈ *X* via *u* ∈ *U* iff (*x*_1_, *u, x*_2_) ∈ *T*. We use the notation $x_{1}\overset{u}{\to }x_{2}$ to mean that (*x*_1_, *u, x*_2_) ∈ *T*. Our notion of transition system is often called a *labeled* transition system in the literature, because each potential transition has a name or label, *u* ∈ *U*. However, we drop the term “labeled” because in Section 2.5 we will introduce a version of transition systems in which the states are relabeled, thereby introducing a new kind of labeling. Note that when working with such transition systems as modeling agency, we are safely within the realm of the Developmental-Explanatory Thesis (EA2). The following definitions are standard (although we do not restrict *X* to be finite):

**Definition 2.2.** Let X=(X,U,T) and $X^{\prime }=\left(X^{\prime },U^{\prime },T^{\prime }\right)$ be transition systems. An *isomorphism* is a bijective function *f*:*X* → *X*′ such that for all *x*_1_, *x*_2_ ∈ *X* and *u* ∈ *U* we have $\left(x_{1},u,x_{2}\right)\in T\Leftrightarrow \left(f\left(x_{1}\right),u,f\left(x_{2}\right)\right)\in T^{\prime }$. A *simulation* is a relation *R* ⊆ *X* × *X*′ such that for all $\left(x_{1},x_{1}^{\prime }\right)\in R$, all *u* ∈ *U* and all *x*_2_ ∈ *X*, we have that if (*x*_1_, *u, x*_2_) ∈ *T*, then there exists $x_{2}^{\prime }\in X^{\prime }$ with $\left(x_{1}^{\prime },u,x_{2}^{\prime }\right)\in T^{\prime }$ and $\left(x_{2},x_{2}^{\prime }\right)\in R$. A *bisimulation* is a relation *R* such that both *R* and *R*^*T*^ = {(*y, x*):(*x, y*) ∈ *R*} are simulations.

The notation $X≅X^{\prime }$ means that X, $X^{\prime }$ are isomorphic, and $X\sim X^{\prime }$ means that there is a bisimulation *R* such that *X* = dom(*R*) and *X*′ = ran(*R*). We speak of *automorphism* and *autobisimulation*, if $X=X^{\prime }$.

We are ready to make the first observation:

**Proposition 2.3.** *If* $X≅X^{\prime }$, *then* $X\sim X^{\prime }$.

*Proof*. Let *f* be an isomorphism *f*:*X* → *X*′. Then $R=\left\{\left(x_{1},x_{2}\right)\in X\times X^{\prime }|x_{2}=f\left(x_{1}\right)\right\}$ is a bisimulation.               □

### 2.1. Transition systems as a unifying concept

There are several ways in which transition systems and their relatives appear in the literature relevant to us.

**Examples 2.4.** Let (*X, U, T*) be a transition system.

1. Let *x*_0_ ∈ *X* and *F* ⊆ *X*. Let $\hat{T}:X\times U\to P\left(X\right)$ be defined by $\hat{T}\left(x,u\right)=\left\{x_{2}\in X|x_{1}\overset{u}{\to }x_{2}\right\}$. Then $\left(X,U,\hat{T},x_{0},F\right)$ is a *nondeterministic automaton*. If in addition *X* and *U* are finite, then it is a *nondeterministic finite automaton* (NFA).
2. Let $\tilde{T}:X\times X\to P\left(U\right)$ be the function $\tilde{T}\left(x_{1},x_{2}\right)=\left\{u\in U|x_{1}\overset{u}{\to }x_{2}\right\}$. Then $\tilde{T}\left(x_{1},x_{2}\right)$ is the set of all *u* that take *x*_1_ to *x*_2_. Then, $\left(X,\tilde{T}\right)$ is a labeled directed graph in which the labels are subsets of *U*. Another way to think of it is as a labeled directed multigraph: the multiplicity of the edge from *x*_1_ to *x*_2_ is $n=|\tilde{T}\left(x_{1},x_{2}\right)|$ and these *n* edges are labeled by the labels from the set $\tilde{T}\left(x_{1},x_{2}\right)$.
3. If for all *x*_1_ ∈ *X* and *u* ∈ *U* there is a unique *x*_2_ ∈ *X* with $x_{1}\overset{u}{\to }x_{2}$, let τ:*X* × *U* → *X* be the function defined such that τ(*x*_1_, *u*) = *x*_2_ iff $x_{1}\overset{u}{\to }x_{2}$. Let *x*_0_ ∈ *X* and *F* ⊆ *X*. Then (*X, U*, τ, *x*_0_, *F*) is a *deterministic automaton*, and if *X* and *U* are finite, then it is a *deterministic finite automaton* (DFA). Without *F*, (*X, U*, τ, *x*_0_) also satisfies the definition of the *temporal filter* of LaValle (2012, 4.2.3). In this case *X* is the *information space* or the *I-space* (usually denoted by I instead of *X*), and *U* is the observation space (usually denoted by *Y* instead of *U*).

### 2.2. Information spaces and filters

We can reformulate the notion of a *history information space* introduced by LaValle (2006) as follows. In this context, *X* is an external state space that characterizes the robot's configuration, velocity, and environment, *U* is an action space, *f* is a state transition mapping that produces a next state from a current state and action, *h* is a sensor mapping that maps states to observations, and *Y* is a sensor observation space. As in LaValle (2006), for each *x* ∈ *X*, let Ψ(*x*) be a finite set of “nature sensing actions” and for each *x* ∈ *X* and *u* ∈ *U* let Θ(*x, u*) be a finite set of “nature actions.” Let *X*_Ψ_ = {(*x*, ψ) ∣ ψ ∈ Ψ(*x*)} and let *h*:*X*_Ψ_ → *Y* be a “sensor mapping” where *Y* is a set called the “observation space.” Let *X*_Θ_ = {(*x, u*, θ) ∣ θ ∈ Θ(*x, u*)} and let *f*:*X*_Θ_ → *X* be the “transition function.” The following definition is an adaptation from LaValle (2006).

**Definition 2.5.** A *valid history I-state for* *X*, Ψ, Θ, *f* is a sequence (*u*_0_, *y*_0_, …, *u*_*k*−1_, *y*_*k*−1_) of length 2*k* for which there exist $\bar{x}=\left(x_{0},\ldots ,x_{k-1}\right)$, $\bar{\psi }=\left(\psi_{0},\ldots ,\psi_{k-1}\right)$ and $\bar{\theta }=\left(\theta_{0},\ldots ,\theta_{k-2}\right)$ such that for all *i* < *k* we have

1. θ_*i*_ ∈ Θ(*x*_*i*_, *u*_*i*_),
2. if *i* < *k* − 1, then *x*_*i* + 1_ = *f*(*x*_*i*_, *u*_*i*_, θ_*i*_),
3. ψ_*i*_ ∈ Ψ(*x*_*i*_),
4. *y*_*i*_ = *h*(*x*_*i*_, ψ_*i*_).

In this case we say that (*u*_0_, *y*_0_, …, *u*_*k*−1_, *y*_*k*−1_) is *witnessed* by $\bar{x}$, $\bar{\psi }$ and $\bar{\theta }$.

Now let I be the set of all valid history I-states for *X*, Ψ, Θ, *f*. For all *k* ∈ ℕ, all $\bar{x}\in X^{k-1}$, all $\bar{\psi }=\left(\psi_{0},\ldots ,\psi_{k-1}\right)$ and all $\bar{\theta }=\left(\theta_{0},\ldots ,\theta_{k-2}\right)$, let $I^{k}\left(\bar{x},\bar{\psi },\bar{\theta }\right)$ be the set of all valid paths (*u*_0_, *y*_0_, …, *u*_*k*−1_, *y*_*k*−1_) witnessed by $\bar{x}$, $\bar{\psi }$, and $\bar{\theta }$. Now let T⊆I×(U×Y)×I be defined by


$$\begin{array}{l} T=\{\left(\eta ,\left(u,y\right),\eta^{\prime }\right)|\text{there exist}k\in \mathbb{N},\\ \bar{x}=\left(x_{0},\ldots ,x_{k-1}\right),\bar{\psi }=\left(\psi_{0},\ldots ,\psi_{k-1}\right),\\ \bar{\theta }=\left(\theta_{0},\ldots ,\theta_{k-2}\right),\theta \in \Theta \left(x_{k-1},u\right)\text{ and }\psi \in \Psi \left(f\left(x_{k-1},u,\theta \right)\right)\\ \text{such that}\\ \eta \in I^{k}\left(\bar{x},\bar{\psi },\bar{\theta }\right)\wedge \eta^{\prime }\in I^{k+1}\left({\bar{x}}^{\prime },{\bar{\psi }}^{\prime },{\bar{\theta }}^{\prime }\right),\\ \text{where }{\bar{x}}^{\prime }={\bar{x}}^{⌢}\left(f\left(x_{k-1},u,\theta \right)\right),\;{\bar{\psi }}^{\prime }=\psi^{⌢}\left(\psi \right),\text{ and }{\bar{\theta }}^{\prime }=\theta^{⌢}\left(\theta \right)\}. \end{array}$$

Here, *x*^⌢^*y* is the concatenation of sequences *x* and *y*. Then (I,U×Y,T) is the *history I-space transition system*.

Suppose for each *x, y* ∈ *X* there is at most one *u* ∈ *U* with $x\overset{u}{\to }y$. Let


$$E_{T}=\left\{\left(x,y\right)\in X^{2}|\exists u\in U\left(x\overset{u}{\to }y\right)\right\},$$

and let *l*:*E*_*T*_ → *U* be defined so that *l*((*x, y*)) is the unique *u* such that $x\overset{u}{\to }y$. Then (*X, E*_*T*_, *l, x*_0_) with *x*_0_ ∈ *X* is a passive I-state graph as in O'Kane and Shell (2017, Def 1).

The following definition is more of a notational than mathematical value.

**Definition 2.6.** Let X=(X,U,T) be a transition system. If for all (*x, u*) ∈ *X* × *U* there is a unique *y* ∈ *X* with (*x, u, y*) ∈ *T*, then we denote the function (*x, u*) ↦ *y* by τ, and write (*X, U*, τ) instead of (*X, U, T*). In this case we call X an *automaton*. Note that usually in computer science literature an automaton is finite and also has an initial state and a set of accepting states, but we do not have those in our definition.

For automata we also use the notation *x* * *u* = τ(*x, u*) and if ū = (*u*_0_, …, *u*_*k*−1_), then *x* * ū is defined by induction for *k* > 1 as follows: *x* * (*u*_0_, …, *u*_*k*−1_) = (*x* * (*u*_0_, …, *u*_*k*−2_)) * *u*_*k*−1_.

**Examples 2.7.** Automata and transition systems can model agent-environment and related dynamics.

1. If (*X*, ·) is a group, *U* ⊆ *X* is a set of generators, and τ(*x, u*) = *x* · *u*, then (*X, U*, τ) is an automaton. For example, consider the situation in which *X* = ℤ × ℤ and *U* = {*a, b, a*^−1^, *b*^−1^} in which *a* = (1, 0) and *b* = (0, 1). Thus, *X* is presented with generators *a*, *b*, and relation *a*·*b* = *b*·*a*. This models an agent moving without rotation in an infinite 2D-grid and the agent can move left, right, up and down. There are no obstacles. The standard Cayley graph is equivalent to the graph based representation of the automaton.
2. Let U^*^ be the set of all finite sequences (“strings”) of elements of *U*. If $ū=\left(u_{0},\ldots ,u_{k-1}\right)\in U^{*}$ and *u*_*k*_ ∈ *U*, we denote by ${ū}^{⌢}u_{k}$ the *concatenation* (*u*_0_, …, *u*_*k*−1_, *u*_*k*_). If ${ū}_{0},{ū}_{1}\in U^{*}$, then ${ū}_{0}⌢{ū}_{1}$ is similarly the concatenation of two strings. The operation of concatenation turns U^*^ into a monoid. Suppose τ:*X* × U^*^ → *X* is an action of the monoid U^*^ on *X* meaning that it satisfies τ(τ(*x*, ū), ū′) = τ(*x*, ū^⌢^ū′) and τ(*x*, ∅) = *x*. Then the automaton (*X, U*, τ) is a discrete-time control system. A sequence of *controls* ū = (*u*_0_, …, *u*_*k*−1_) produces a unique *trajectory* (*x*_0_, …, *x*_*k*_), given the initial state *x*_0_ by induction: *x*_*i*+1_ = τ(*x*_*i*_, *u*_*i*_) for all *i* < *k*.
3. Consider an automaton (*X, U*, τ) in which *U* is a group, and τ is a group action of *U* on *X*. In some situations it can be natural to consider the set of motor-outputs of an agent to be a group: the neutral element is no motor-output at all, every motor-output has an “inverse” for which the effect is the opposite, or negating (say, moving right as opposed to moving left), the composition of movements is many movements applied consecutively. The action τ of *U* on *X* is then the realization of those motor-outputs in the environment. In realistic scenarios, however, this is not a good way to model the sensorimotor interaction because of the following reason. Suppose the agent has actions “left” and “right,” but it is standing next to an obstacle on its left. Then moving “left” will result in staying still (because of the obstacle), but moving “right” will result in actually moving right, if there is no obstacle at the right of the agent. In this situation the sequence “left-right” results in a different position of the agent than the sequence “right-left,” so if “left” and “right” are each other's inverses in *G*, then the axioms of group action are violated.
4. Note that if *T* = ∅, then (*X, U, T*) is a transition system.
5. Let *X* = {0, 1}^*^ as in (2), *U* = {0}, and (*x*, 0, *y*) ∈ *T* if and only if |*y*| = |*x*| + 1, then (*X, U, T*) is a transition system, where |*x*| is the length of the string *x*.
6. If (*X, U, T*) is a transition system and *E* ⊆ *X* an equivalence relation, then (*X*/*E, U, T*/*E*) is a transition system, where *X*/*E* = {[*x*]_*E*_ ∣ *x* ∈ *X*} and *T*/*E* = {([*x*]_*E*_, *u*, [*y*]_*E*_) ∣ (*x, u, y*) ∈ *T*}, and / denotes a quotient space; see Definition 2.33.

### 2.3. Sensorimotor systems

Next, we will define a *sensorimotor system*, which is a special case of a transition system. Following the tenet (EA1) that “environment is inseparable from the body which is inseparable from the brain,” our sensorimotor systems can model any part of the environment-body-brain coupling. The model that describes the environment differs from the one that describes the agent merely in the type of structure it possesess, but not in an essential mathematical way.

SM-systems can be thought of as a partial specification of (some part of) the brain-body-environment coupling. Physicalist determinism demands that under full specification^2^ we are left with a deterministic system. A specification is partial when it leaves room for unknowns in some, or all, parts of the system.

**Definition 2.8.** A *sensorimotor system* (or *SM-system*) is a transition system (*X, U, T*) where *U* = *S* × *M* for some sets *S* and *M*, which we call in this context the *sensory set* and the *motor set, respectively*.

The interpretation is that if $x\overset{\left(s,m\right)}{\to }y$, then *s* is the sensation that either occurs at *x*, or along the transition to the next state *y*, and *m* the motor action which leads to the transition. We will show later how SM-systems can be connected together (Definition 2.22) to form coupled systems. Sometimes an SM-system is modeling a brain-body totality, and other times it is modeling body-environment totality. A coupling between these two will model the brain-body-environment totality. This is a flexible framework which enables enactivist-style analysis. We do not assume that the agent “knows” the effect of a given *m* ∈ *M* or that the “meaning” of a given *s* ∈ *S*. The sets *S* and *M* are purely mathematical sets denoting the interface between the agent and the environment from the third person perspective.

In fact, the sensory and motor components can be decoupled which might be more natural from the mathematics' point of view in some cases. The following shows that we can look at it both ways.

**Definition 2.9.** An *asynchronous SM-system* is a transition system (*X, U, T*) such that there exist partitions *U* = *S* ∪ *M* and *X* = *X*_*s*_ ∪ *X*_*m*_ such that for all (*x, u, y*) ∈ *T* we have

1. if *x* ∈ *X*_*s*_, then *u* ∈ *S*,
2. if *x* ∈ *X*_*m*_, then *u* ∈ *M*, and
3. *x* ∈ *X*_*m*_ ⇔ *y* ∈ *X*_*s*_.

Thus, the state space of a sequential SM-system contains separate *sensory states* and *motor states*.

**Definition 2.10.** Suppose *E* is an equivalence relation on a set *X*. We say that a map *f*:*X* → *X* is *E-preserving* if for all *x, y* ∈ *X*, we have *xEy* ⇔ *f*(*x*)*Ef*(*y*).

There is a natural correspondence between SM-systems and their asynchronous counterpart:

**Theorem 2.11.** *Let* SM *and* aSM *be the classes of SM-systems and asynchronous SM-systems, respectively. There are functions F*:SM → aSM *and G*:aSM → SM *such that*

1. *F and G are isomorphism and bisimulation preserving*,
2. *restricted to finite systems, *F* and *G* are polynomial-time computable, and restricted to the infinite ones they are Borel-functions in the sense of classical descriptive set theory (Kechris, 1994)*.

*Proof*. See Appendix B.               □

Another type of a system, which is in a similar way equivalent to a special case of an SM-system, is a state-labeled transition system which we will introduce next, and prove a similar result, Lemma 2.19.

### 2.4. Quasifilters and quasipolicies

The amount of information specified in a given SM-system depends on which part of the brain-body-environment system we are modeling. At one extreme, we specify the environment's dynamics down to the small detail and leave the brain's dynamics completely unspecified. In this case the SM-system will have only one sensation corresponding to each state and the transition to the next state will be completely determined by knowing the motor action. This is, in a sense, the environment's perspective. At the other extreme, we specify the brain completely, but leave the environment unspecified. We “don't know” which sensation comes next, but we “know” which motor actions are we going to apply. This is in a sense the perspective of the agent. The first extreme case is the perspective often taken in robotics and other engineering fields when either specifying a planning problem (Ghallab et al., 2004; Choset et al., 2005; O'Kane and LaValle, 2008), or designing a filter (Hager, 1990; Thrun et al., 2005; LaValle, 2012; Särkkä, 2013) (also known as sensor fusion). This is why we call SM-systems of that sort *quasifilters* (Definition 2.12). The other extreme is the perspective of a policy. The policy depends on sensory input, but the motor actions are determined (by the policy). This is why we call the SM-systems of the latter sort *quasipolicy*. The “quasi-” prefix is used because both are weaker and more general notions than those that appear in the literature; see Remarks 2.20 and 2.21.

Another way to look at this is the dichotomy between virtual reality (VR), and robotics. In virtual reality, scientists are designing the (virtual) environment for an agent whereas in robotics they are typically designing an agent for an environment. In the former case the agent is partially specified: the type of embodiment is known (*S* and *M* are known) and some types of patterns of embodiment are known (eye-hand coordination). However, the specific actions to be taken by the agents are left unspecified. The job of the designer is to specify the environment down to the smallest detail, so that every sequence of motor actions of the agent yields targeted sensory feedback. The VR-designer is designing a quasifilter constrained by the partial knowledge of the agent's embodiment and internal dynamics. The case for the robot designer is the opposite. She has a partial specification of the robot's intended environment and usually works with a complete specification of the robot's mechanics. She is designing a quasipolicy. For VR-designers the agent is a black box; for roboticists the agent is a white box (Suomalainen et al., 2020) (unless the task is to reverse engineer an unknown robot design). For the environment, the roles are reversed. A similar dichotomy can be seen between biology (in which the agent is a black box) and robotics (in which it usually is a white box).

All the definitions in this section are new.

**Definition 2.12.** Suppose that (*X, S* × *M, T*) is an SM-system with the property that for all *x*_1_ ∈ *X* there exists *s*_*x*_1__ ∈ *S* such that for all *x*_2_ ∈ *X* and all (*s, m*) ∈ *S* × *M* we have that $x_{1}\overset{\left(s,m\right)}{\to }x_{2}$ implies *s* = *s*_*x*_1__. Then, (*X, S* × *M, T*) is a *quasifilter*.

In a quasifilter the sensory part of the outgoing edge is unique. The dual notion (quasipolicy) is when the motor part is unique:

**Definition 2.13.** Suppose that (*X, S* × *M, T*) is an SM-system with the property that for all *x* ∈ *X* there exists *m*_*x*_ ∈ *M* such that for all *y* ∈ *X* and all (*s, m*) ∈ *S* × *M* we have that $x\overset{\left(s,m\right)}{\to }y$ implies *m* = *m*_*x*_. Then, (*X, S* × *M, T*) is a *quasipolicy*.

Before explaining the connections between quasifilter and a filter and quasipolicy and a policy, let us define projections of the sensorimotor transition relation to “motor” and to “sensory”:

**Definition 2.14.** Given an SM-system (*X, S* × *M, T*), let


$$\begin{array}{l} T_{M}=\left\{\left(x,m,y\right)\in X\times M\times X|\exists s\in S\left(x,\left(s,m\right),y\right)\in T\right\}\\ \;T_{S}=\left\{\left(x,s,y\right)\in X\times S\times X|\exists m\in M\left(x,\left(s,m\right),y\right)\in T\right\}. \end{array}$$

These are called the *motor* and the *sensory projections*, respectively of the sensorimotor transition relation. They are also called the *motor transition relation* and the *sensory transition relation*, respectively. The corresponding transition systems (*X, M, T*_*M*_) and (*X, S, T*_*S*_) are called the *motor* and the *sensory projection systems*.

**Definition 2.15.** Given a transition system (*X, U, T*), and *x* ∈ *X*, let *O*_*T*_(*x*) ⊆ *U* be defined as the set $O_{T}\left(x\right)=\left\{u\in U|\left(\exists y\in X\right)\left(x\overset{u}{\to }y\right)\right\}$. Combining this notation with the one introduced in Example 2.4(2), given *x, y* ∈ *X*, we have


$$O_{T}\left(x\right)=\underset{y\in X}{⋃}\tilde{T}\left(x,y\right).$$

For a transition relation *T* ⊆ *X* × (*S* × *M*) × *X*, define its *transpose* by *T*^*t*^ ⊆ *X* × (*S* × *M*) × *X* such that *T*^*t*^ = {(*x*, (*m, s*), *y*) ∣ (*x*, (*s, m*), *y*) ∈ *T*}. Note that (*T*^*t*^)^*t*^ = *T*. For a subset of a Cartesian product *A* ⊆ *S* × *M*, let *A*_1_ be the projection to the first coordinate *A*_1_ = {*s* ∈ *S*∣(∃*m* ∈ *M*)((*s, m*) ∈ *A*)} and *A*_2_ the projection to the second one: *A*_2_ = {*m* ∈ *M*∣(∃*s* ∈ *S*)((*s, m*) ∈ *A*)}.

Mathematically coupling of two transition systems is symmetric [see Theorem 2.24(3)], but from the cognitive perspective there is (usually) an asymmetry between the agent and the environment (which can be evident from some specific properties of the agent and of the environment). Because of the partial symmetry, many properties of an agent can dually be held by the environment and vice versa. The following proposition highlights the duality between quasipolicy and quasifilters: reversing the roles of the environment and the agent.

**Proposition 2.16.** *For an SM-system* X=(X,S×M,T) *the following are equivalent*:

1. X *is a quasifilter*,
2. $X^{t}=\left(X,S\times M,T^{t}\right)$ *is a quasipolicy*,
3. $O_{T_{S}}={\left(O_{T}\left(x\right)\right)}_{2}={\left(O_{T^{t}}\left(x\right)\right)}_{1}$ *is a singleton for each x* ∈ *X*.

*Similarly*, X *is a quasipolicy if and only if O*_*T*_*M*__(*x*) = (_*O*_*T*_(*X*))1_ is a singleton for each *x* ∈ *X*.

*Proof*. A straightforward consequence of all the definitions.               □

### 2.5. State-relabeled transition systems

It will become convenient in the coming framework to assign labels to the states. The elements *x* of the state space *X* are already named; thus, our labeling can be more properly considered as a *relabeling via* a function *h*:*X* → *L*, in which *L* is an arbitrary set of *labels*. This allows partitions to be naturally induced over *X* by the preimages of *h*. Intuitively, this will allow the state space *X* to be characterized at different levels of “resolution” or “granularity.” Thus, we have the following definition:

**Definition 2.17.** A *state-relabeled transition system* (or simply *labeled transition system*) is a quintuple (*X, U, T, h, L*) in which *h*:*X* → *L* is a labeling function and (*X, U, T*) is a transition system.

We think of *state-relabeled* to be a more descriptive term, but we shorten it in the remainder of this paper to being simply *labeled*.

*Remark* 2.18. In an analogy to Definition 2.6, a labeled transition system is a *labeled automaton*, if *T* happens to be a function; in other words, for all (*x, u*) ∈ *X* × *U* there is a unique *y* ∈ *X* with (*x, u, y*) ∈ *T*. In this case we may denote this function by τ:(*x, u*) ↦ *y* and work with the labeled automaton (*X, U*, τ, *h, L*). For example, the temporal filter in Section 2.1 is a labeled automaton.

The isomorphism and bisimulation relations are defined similary as for transition systems, but in a label-preserving way.

One intended application of a labeled transition system (*X, U, T, h, L*) is that *h* is a sensor mapping, *L* is a set of sensor observations, and *U* is a set of actions. Thus, actions *u* ∈ *U* allow the agent to transition between states in *X* while *h* tells us what the agent senses in each state. We intend to show that this can be seen as a special case of an SM-system by proving a theorem similar to Theorem 2.11, but stronger, namely these corresponces preserve isomorphism:

**Lemma 2.19**. *Let* F *be the class of quasifilters*, P *the class of quasipolicies, and* L *the class of labeled systems. Then there are one-to-one maps*


$${\mathrm{LTS}}_{P}:P\to L\text{ and }{\mathrm{LTS}}_{F}:F\to L$$

*such that*

1. LTS_*P*_ *and* LTS_*F*_ *are isomorphism and bisimulation preserving*,
2. *restricted to finite systems*, LTS_*P*_ *and* LTS_*F*_ *are polynomial-time computable, and restricted to the infinite ones they are Borel-functions in the sense of classical descriptive set theory*.

*Proof*. See Appendix B               □

*Remark* 2.20. Let X=(X,S×M,T) be a quasifilter and $X^{\prime }={\mathrm{LTS}}_{F}\left(X\right)=\left(X,M,T_{M},h,S\right)$ as in Lemma 2.19. Suppose further that for each *x, y* ∈ *X* there is at most one *u* ∈ *U* with $x\overset{u}{\to }y$. Let


$$E_{T}=\left\{\left(x,y\right)\in X^{2}|\exists u\in U\left(x\overset{u}{\to }y\right)\right\},$$

Then (*X, M, E*_*T*_, *x*_0_) coincides with the definition of a filter (O'Kane and Shell, 2017, Def 3). If it is also an automaton, meaning that above we replace “at most one” by “exactly one,” then every sequence of motor actions (*m*_0_, …, *m*_*k*−1_) determines a unique resulting state *x*_*k*−1_ ∈ *X*. This is analogous, and can be proved in the same way, as the fact that each sequence of sensory data determines a unique resulting state in Remark 2.21 below.

*Remark* 2.21. Usually, a *policy* is a function which describes how an agent chooses actions based on its own past experience. Thus, if *M* is the set of motor commands and *S* is the set of sensations, a policy is a function π:S^*^ → *M* where S^*^ is the set of finite sequences of sensory “histories”; see for example (LaValle, 2006). Now, suppose that an SM-system X=(X,S×M,T) is a quasipolicy in the sense of Definition 2.13 and let *x* ↦ *m*_*x*_ be as in that Definition. Assume further that X is an automaton (Section 2.1) and let τ:*X* × (*S* × *M*) → *X* be the corresponding transition function so that for all *x* ∈ *X* and (*s, m*) ∈ *S* × *M* we have (*x*, (*s, m*), τ(*x*, (*s, m*))) ∈ *T*. Let *x*_0_ ∈ *X* be an initial state. We will show how the pair $\left(X,x_{0}\right)$ defines a function π:S^*^ → *M* in a natural way. Let $\bar{s}=\left(s_{0},\ldots ,s_{k-1}\right)\in S^{k}$ be a sequence of sensory data. If *k* = 0, and so $\bar{s}=\left(\;\right)=\emptyset$, let $\pi \left(\bar{s}\right)=m_{x_{0}}$. If *k* > 0, assume that π(*s*_0_, …, *s*_*k*−2_) and *x*_*k*−1_ are both defined (induction hypothesis). Then let *x*_*k*_ = τ(*x*_*k*−1_, (*m*_*x*_*k*−1__, *s*_*k*−1_)) and π(*s*_0_, …, *s*_*k*−2_, *s*_*k*−1_) = *m*_*x*_*k*__. The idea is that because of the uniqueness of *m*_*x*_, a sequence of sensory data determines (given an initial state) a unique path through the automaton X.

### 2.6. Couplings of transition systems

The central concept of this work pertaining to all principles (EA1)–(EA5) is the coupling of SM-systems. We define coupling, however, for general transition systems with the understanding that our most interesting applications will be for SM-systems where *U*_0_ = *U*_1_ = *S* × *M*. The idea is that in every transition there is a sensory component and a motor component. The set *S* could be thought of as all possible events that trigger afferent nervous signals, or their combinations. The elements of *M* are those events that are triggered by efferent nervous signals. This is an abstract space and in transitioning from one state to another some subset of *S* × *M* is “active.” If we know little of what kind of sensory data the agent receives during the transition, then that transition will occupy a subset of *S* × *M* whose projection to the *S*-coordinate is large. If, on the other hand we know a lot, and can specify the exact sensory data, then the projection to the *S*-coordinate is small. Vice versa, if we do not know which motor actions lead from one state to another, then the projection of the corresponding subset to the *M*-coordinate is large etc. This was made more precise in Section 2.4. The fact that the transition consists of pairs (*s, m*) where *s* is a sensory input and *m* is a motor command does not mean that the agent is equipped with the semantics of what *m* “means,” or what it “does” in the world. The effect of *m* is “computed” by the environment and the agent only receives the next “*s*” as the feedback. It might have been more intuitive, but more cumbersome to make this definition in terms of functions that map events of the environment to sensory stimuli and internal events of the nervous system to motor actions, and further functions that map the motor actions to the actual events in the environment, etc., but from the point of view of essential mathematical structure these extra identifications wouldn't add anything qualitatively new.

**Definition 2.22.** Let $X_{0}=\left(X_{0},U_{0},T_{0}\right)$ and $X_{1}=\left(X_{1},U_{1},T_{1}\right)$ be two transition systems. The *coupled* system $X_{0}*X_{1}$ is the transition system (*X, U, T*) defined as follows: *X* = *X*_0_ × *X*_1_, *U* = *U*_0_ ∩ *U*_1_, and


$$\begin{array}{l} T=T_{0}*T_{1}=\{((x_{0},x_{1}),u,(y_{0},y_{1}))|(x_{0},u,y_{0})\\ \in T_{0}\wedge (x_{1},u,y_{1})\in T_{1}\}. \end{array}$$

Equivalently, for all $\left(\left(x_{0},x_{1}\right),\left(y_{0},y_{1}\right)\right)\in {\left(X_{0}\times X_{1}\right)}^{2}$ we have


$$\tilde{T}\left(\left(x_{0},y_{0}\right),\left(x_{1},y_{1}\right)\right)={\tilde{T}}_{0}\left(x_{0},x_{1}\right)\cap {\tilde{T}}_{1}\left(y_{0},y_{1}\right)$$

(recall the $\tilde{T}$ notation from Example 2.4(2)).

**Figure 2:**
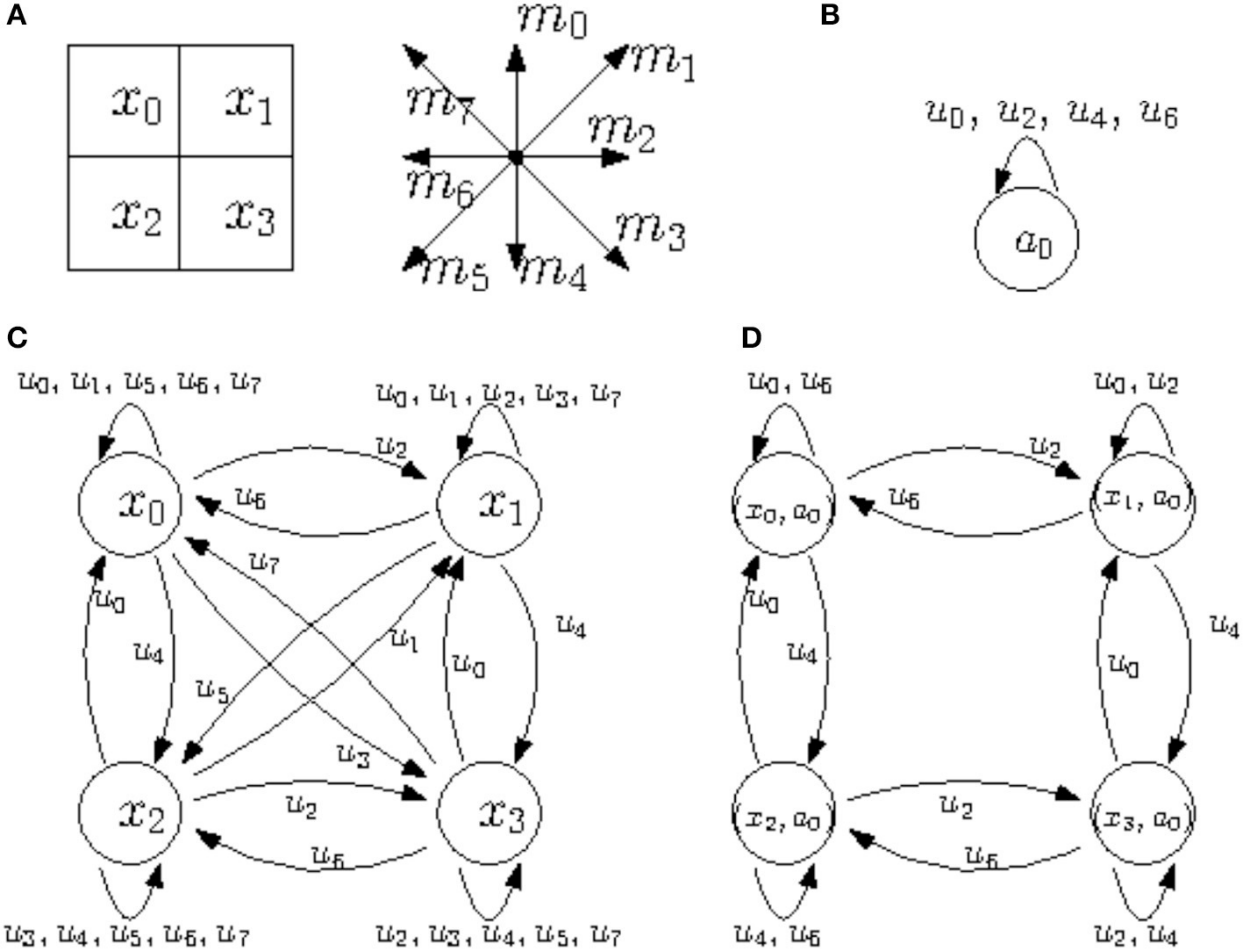
**(A)** States and actions for the transition system $X_{0}$ that describes a 2-by-2 grid. 8 actions populating the set *M* = {*m*_0_, …, *m*_7_} correspond to a move (to a neighbor cell if possible) either sideways or diagonally. Suppose *S* is a singleton such that *S* = {*s*}. Then, in the following, *u*_*i*_ corresponds to the transition *u*_*i*_ = (*m*_*i*_, *s*) for *i* = 1, …, 7. **(B)** Transition system $X_{1}$. **(C)** Transition system $X_{0}$. **(D)** The coupled system $X_{0}*X_{1}$.

**Example 2.23.** A simple example of coupling is illustrated in Figure 2.

Mathematically the coupling is a product of sorts. If we think of one transition system as “the environment” and the other as “the agent,” then the coupling tells us about all possible ways in which the agent can engage with the environment. The fact that the state space of the coupled system is the product of the state spaces of the two initial systems reflects the fact that the coupled system includes information of “what would happen” if the environment was in any given state while the agent is in any given (“internal”) state.

We immediately prove the first theorem concerning coupling:

**Theorem 2.24.** *Suppose that* $X_{i}=\left(X_{i},U_{i},T_{i}\right)$ *and* $X_{i}^{\prime }=\left(X_{i}^{\prime },U_{i}^{\prime },T_{i}^{\prime }\right)$ *for i* ∈ {0, 1} *are four SM-systems. Then the following hold*:

1. *If* $X_{i}≅X_{i}^{\prime }$ *for i* ∈ {0, 1}, *then* $X_{0}*X_{1}≅X_{0}^{\prime }*X_{1}^{\prime }$.
2. *If* $X_{i}\sim X_{i}^{\prime }$ *for i* ∈ {0, 1}, *then* $X_{0}*X_{1}\sim X_{0}^{\prime }*X_{1}^{\prime }$.
3. $X_{0}*X_{1}≅X_{1}*X_{0}$.

*Proof*. See Appendix B               □

Coupling provides an interesting way to compare SM-systems from the “point of view” of other SM-systems. For example, given an SM-system E one can define an equivalence relation on SM-systems by saying that $I\sim_{E}I^{\prime }$, if $E*I=E*I^{\prime }$. If E is the “environment” and I, $I^{\prime }$ are “agents,” this is saying that the agents perform identically in this particular environment. Or vice versa, for a fixed I, the relation $E*I=E^{\prime }*I$ means that the environments are indistinguishable by the agent I.

*Remark* 2.25. In the definition of coupling we see that the two SM-systems constrain each other. This is seen from the fact that in the definition we take intersections. For example, when an agent is coupled to an environment, it chooses certain actions from a large range of possibilities. In this way the agent structures its own world through the coupling (EA3). To make this notion further connect to enactivist paradigm, we invoke the dynamical systems approach to cognition (Tschacher and Dauwalder, 2003). An *attractor* in a transition system X=(X,U,T) is a set *A* ⊆ *X* with the property that for all infinite sequences


$$x_{0}\overset{u_{0}}{\to }x_{1}\overset{u_{1}}{\to }\cdots x_{k-1}\overset{u_{k-1}}{\to }x_{k}\overset{u_{k}}{\to }\cdots$$

there are infinitely many indices *n* such that *x*_*n*_ ∈ *A*. There could be other possible definitions, such as “for all large enough *n*, *x*_*n*_ ∈ *A*”. For the present illustration purposes it is, however, irrelevant. It could be the case that *A* ⊆ *X* is not an attractor of X, but after coupling with $X^{\prime }=\left(X^{\prime },U^{\prime },T^{\prime }\right)$, *A* × *X*′ may be an attractor of $X*X^{\prime }$. Thus, if X is the environment and $X^{\prime }$ is the agent and *A* is a set of desirable environmental states, then we may say that the agent is well attuned to X, if *A* was not initially an attractor, but in $X*X^{\prime }$, then *A* × *X*′ becomes one. It could also be that the agent needs to arrive to *A* while being in a certain type of an internal state *B* ⊆ *X*′, for example, if *A* is “food” and *B* is “hungry”. Then it is not important that *A* × *X*′ is an attractor, but it is imperative that *A* × *B* is one.

### 2.7. Unconstrained and fully constrained SM-systems

As we mentioned before, the information specified in an SM-system depends on which part of the brain-body-environment system we are modeling. In the extreme case we do not specify *anything*, except for the very minimal information. Consider a body of a robot for which the set of possible actions (or motor commands) is *M* and the set of possible sensor observations is *S*. Suppose that is all we know about the robot. We do not know what kind of environment it is in and we do not know what kind of “brain” (a processor or an algorithm) it is equipped with. Thus, we do not know of any constraints the robot may have in sensing or moving. We then model this robot as an *unconstrained* SM-system:

**Definition 2.26.** An SM-system (*X, S* × *M, T*) is called *unconstrained* iff for all *x* ∈ *X*, we have *O*_*T*_(*x*) = *S* × *M*; recall Definition 2.15.

Unconstrained systems have the role of a neutral element with respect to coupling (Proposition 2.29). We now show that given all unconstrained SM-systems with shared *M* and *S* are mutually bisimulation equivalent:

**Proposition 2.27.** *Suppose that* X=(X,S×M,T) *and* $X^{\prime }=\left(X^{\prime },S\times M,T^{\prime }\right)$ *are unconstrained systems. Then* $X\sim X^{\prime }$.

*Proof*. See Appendix B               □

There are many intuitions behind the above. An unconstrained system is one where anything could happen: the agent might perform any actions in any order and the environment could provide the agent with any sensory data. Such a world is reminiscent of *white noise*. Such a system is only interesting from an abstract mathematical perspective, it is in some sense “maximal”. The content of Proposition 2.27 is that such systems are indistinguishable from each other. An unconstrained system has a similar role with respect to all SM-systems as the free group has to other groups, although we haven't made this universality claim precise in the present paper. Intuitively it means that every possible agent-environment combination can be found as a subsystem (or possibly a quotient) of the unconstrained one. The term “unconstrained” refers in particular to that when coupled to other systems, this system doesn't constrain them, so it acts in the same way as 0 in arithmetic addition (Proposition 2.29). The opposite is the fully constrained system (Definition 2.31, Proposition 2.32). In that case, the intuition is the opposite: in environments where nothing happens and actions do not have any effects, any agent is as good as any other and vice versa: agents that don't do anything are equivalent.

**Corollary 2.28.** *The SM-system ε* = ({0}, {0} × (*S* × *M*) × {0}}) *is the unique, up to bisimulation, unconstrained system*.

**Proposition 2.29.** *Let ε be as in Corollary 2.28 and let* X=(X,S×M,T) *be any SM-system. Then* X*ε≅X.

**Corollary 2.30.** *If* X *and* $X^{\prime }$ *are SM-systems and* $X^{\prime }$ *is unconstrained, then* $X*X^{\prime }\sim X$.

*Proof*. By Corollary 2.28 $X^{\prime }\sim \varepsilon$, So by Theorem 2.24 we have $X*X^{\prime }\sim X*\varepsilon$. However, by Proposition 2.29, X*ε~X; thus, $X*X^{\prime }\sim X$.               □

The opposite of an unconstrained system is a fully constrained one:

**Definition 2.31.** An SM-system (*X, S* × *M, T*) is *fully constrained* iff *T* = ∅.

**Proposition 2.32.** *Dually to the propositions above, we have that (1) all fully constrained systems are bisimulation equivalent to each other, (2) the simplest example being λ = ({0}, *S* × *M*, ∅), and (3) if* X=(X,S×M,T) *is another SM-system, then* X*λ~λ.

All transition systems are in some sense between the fully constrained and the unconstrained, these being the two theoretical extremes.

### 2.8. Quotients of transition systems

When considering labelings and their induced equivalence relations, it will be convenient to develop a notion of quotient systems, analogous to quotient spaces in topology. Suppose X=(X,U,T) is a transition system and *E* is an equivalence relation on *X*. We can then form a new transition system, called the *quotient* of X by *E* in which the new states are *E*-equivalence classes and the transition relation is modified accordingly.

The following definition of a quotient is standard in Kripke model theory, especially bisimulation theory:

**Definition 2.33.** Suppose X=(X,U,T) and *E* are as above. Let *X*/*E* = {[*x*]_*E*_ ∣ *x* ∈ *X*}, in which each [*x*]_*E*_ is an equivalence class of states *x* under relation *E*, and *T*/*E* = {([*x*]_*E*_, *u*, [*y*]_*E*_)∣(*x, u, y*) ∈ *T*}. Then X/E=(X/E,U,T/E) is the *quotient* of (*X, U, T*) by *E*.

The following definition is inspired by the idea of sensory pre-images, see LaValle (2019), but is also needed for technical reasons.

**Definition 2.34.** Given any function *h*:*X* → *L*, denote by *E*^*h*^ the inverse-image equivalence: *E*^*h*^ = {(*x, y*) ∈ *X*^2^ ∣ *h*(*x*) = *h*(*y*)}. We will denote the equivalence classes of *E*^*h*^ by [*x*]_*h*_ instead of ${\left[x\right]}_{E^{h}}$ if no confusion is possible.

The equivalence relation *E*^*h*^ partitions *X* according to the preimages of *h*, as considered in the sensor lattice theory of LaValle (2019). The partition of *X* induced by *h* directly yields an quotient transition system by applying the previous two definitions:

**Definition 2.35.** Let X=(X,U,T) be a transition system and *h*:*X* → *L* be any mapping. Then define X/h to be $X/E^{h}$ where we combine Definitions 2.34 and 2.33.

**Proposition 2.36.** *If h is one-to-one, then* X/h≅X.

*Proof*. *h* is one-to-one if and only if *E*^*h*^ is equality, in which case it is straightforward to verify that the function $x\mapsto {\left[x\right]}_{E^{h}}$ is an isomorphism.               □

For *h*:*X* → *L*, the transition system (*X*/*h, U, T*/*h*) is essentially a new state space over the preimages of *h*. In this case X/h is called the *derived information space* (as used in LaValle, 2006). More precisely:

**Proposition 2.37.** *Let L*′ = *ran*(*h*) ⊆ *L*. *Define*


$$\begin{array}{l} T^{\prime }=\left\{\left(l,u,l^{\prime }\right)\in L^{\prime }\times U\times L^{\prime }|\left(h^{-1}\left(l\right),u,h^{-1}\left(l^{\prime }\right)\right)\in T/h\right\}\\ \;=\left\{\left(h\left(x\right),u,h\left(y\right)\right)|\left(x,u,y\right)\in T\right\}. \end{array}$$

*Then* (*X*/*h, U, T*/*h*) *is isomorphic to* (*L*′, *U, T*′) *via the isomorphism* $f:{\left[x\right]}_{E^{h}}\mapsto h\left(x\right)$.

*Proof*. See Appendix B               □

The intuitive meaning of the quotient is the following. There is a Soviet comedy film from the 1970's where the main character ends up in an apartment in Leningrad, while he thinks that he is actually in Moscow. The apartement in Leningrad is identical to his home in Moscow and he cannot distinguish between them. He thinks for a while that he is at his home in Moscow while being in an apartment in Leningrad. Even his key from Moscow worked for the Leningrad apartment. The pun is that in Soviet times all houses were built according to the same blueprint. Now, before he realized his situation, as far as he was concerned, he *was* in Moscow. He thought he came to the same place in the evening as in the morning, while he actually didn't. The idea of the quotient captures exactly that: We identify those states that “look the same” (the label is the same) even though they are actually different states. In fact, let us look at a cognitive system on several levels of granularity: When I type on my laptop at home or in a cafeteria, my fingers experience the keyboard in (approximately) the same way. As far as my fingers (and associated motor areas) are concerned, we can identify all situations where they are pressing keys on my keyboard. On a higher level, I might be coming home after a 10 h time and experience as if I am in the same place, but we all know that the planet, on which my home is, has moved, so I actually am not in the same place, just like the main character in the movie referenced above.

## 3. Illustrative examples of SM-systems

We next illustrate how sensorimotor systems model body-environment, brain-body, and brain-body-environment couplings. Consider a body in a fully understood and specified deterministic environment. In this case the body-environment system will be modeled by a quasifilter, Definition 2.12. Instead of using the quasifilter definition, we work with a labeled transition system which, according to Proposition 2.19, is equivalent. According to the assumption of full specification, we will in fact work with labeled automata.

The body has a set *M* of possible motor actions each of which has a deterministic influence on the body-environment dynamics. Denote the set of body-environment states by *E*_0_. Whenever a motor action *m* ∈ *M* is applied at a body-environment state *e* ∈ *E*, a new body-environment state *A*(*e, m*) ∈ *E* is achieved. At each state *e* ∈ *E* the body senses data σ(*e*). Denote the set of sensations by *S*. In this way, the labeled automaton $E_{0}=\left(E,M,A,\sigma ,S\right)$ models this body-environment system. This model is ambivalent toward the agent's internal dynamics, its strategies, policies and so on, but not ambivalent toward its embodiment and its environment's structure. In fact, it characterizes them completely.

Alternatively, consider a brain in a body, and suppose that the brain is fully understood and deterministic (for example, perhaps it is designed by us), but we do not know which environment it is in. We model this by an SM-system which is a quasipolicy. Again, by the analogous considerations as above, we work directly an equivalent labeled automaton specification. Denote the set of internal states of the brain by *I*. The agent's internal state is a function of the sensations; therefore, let *B*:*I* × *S* → *I* be a function (*B* stands for *brain*) that takes one internal state to another based on new sensory data. At each internal state, the agent produces a motor output which is an element of the set *M*; therefore, let μ:*I* → *M* be a function assigning a motor output to each internal state. Now, I=(I,S,B,μ,M) is a labeled transition system modeling this agent. It is ambivalent toward the type of the environment the agent is in, but it is not ambivalent toward the agent's internal dynamics, policies, strategies and so on; in fact, it determines them completely.

Now, the coupling of the environment E and the agent A is the SM-system obtained as


$$\begin{array}{l} {\mathrm{LTS}}_{F}^{-1}\left(E\right)*{\mathrm{LTS}}_{P}^{-1}\left(A\right). \end{array}$$

The sensory and motor sets *S* and *M* capture the interface between the brain and the environment because they characterize the body (but not the *embodiment*).

**Example 3.1.** Consider an agent that has four motor outputs, called “up” (*U*), “down” (*D*), “left” (*L*), and “right” (*R*), and there is no sensor feedback (this defines the body). In Corollary 2.28 we gave a minimal example of an unconstrained SM-system. On the other extreme one can give large examples. For instance the free monoid generated by the set *M* = {*U, D, L, R*}.

**Figure 3:**
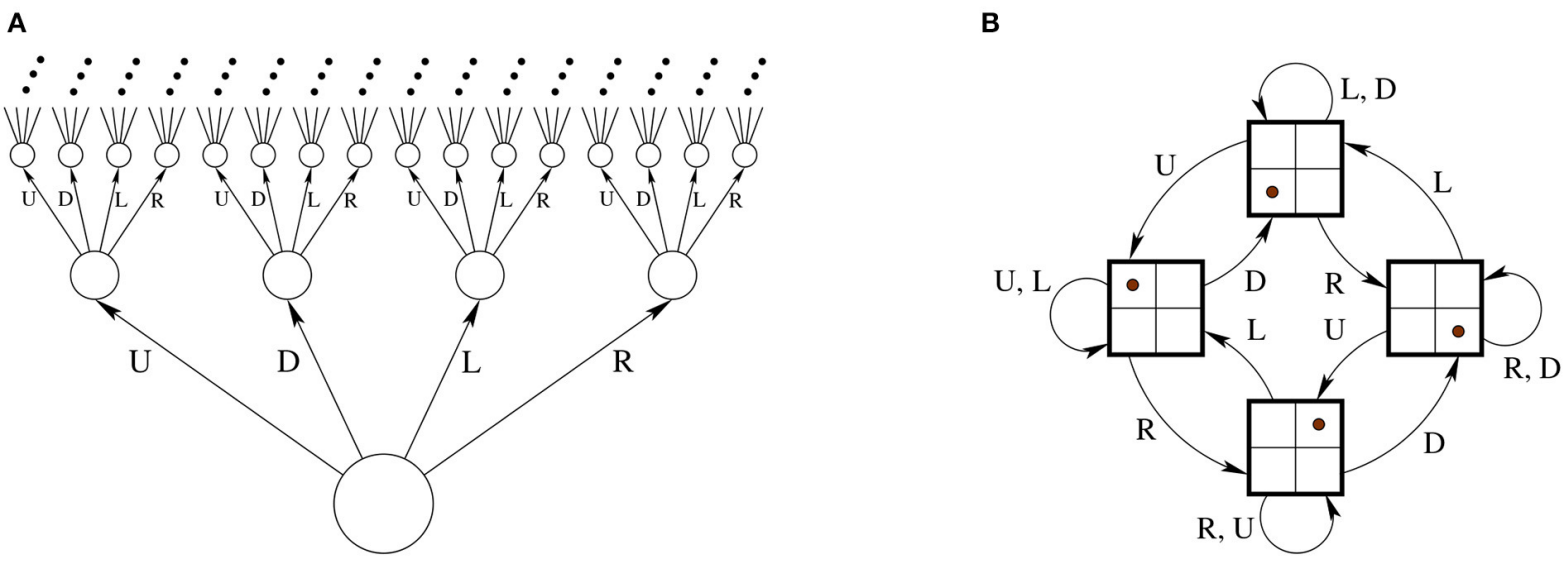
**(A)** Having motor commands and no sensory feedback leads to an infinite tree automaton. **(B)** Once the body is coupled with a 2 × 2 grid environment, a four-state automaton results.

Let *X* be the set of all possible finite strings in the four “letter” alphabet *M*, let *T* = {(*x, m, y*)∣*x*^⌢^*m* = *y*}. “No sensor data” is equivalent to always having the same sensor data; thus, we can assume that *S* = {*s*_0_} is a singleton and the sensor mapping *h*:*X* → *S* is constant.^3^ The resulting unconstrained transition system U=(X,T,M,σ,S) can be represented by an infinite quaternary tree, shown in Figure 3A.

**Figure 4:**
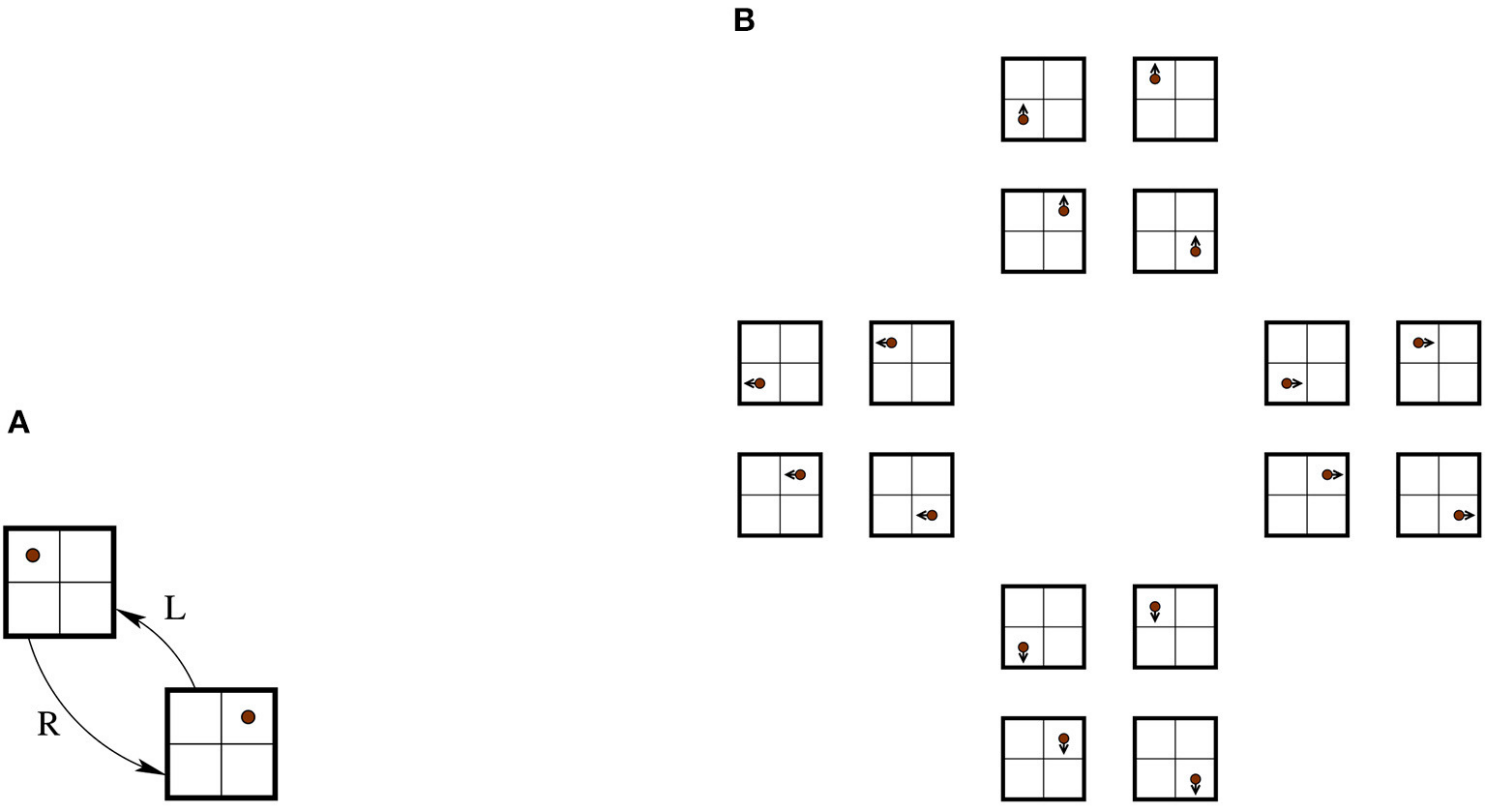
**(A)** A two-state automaton results from the realized policy. **(B)** If there are only two actions (rotate 90 degrees counterclockwise and going straight) then the second automaton has 16 states instead of four as in Figure 3B.

Suppose that this body is situated in a 2 × 2 grid. The body can occupy one of the four grid's squares at a time, and when it applies one of the movements, it either moves correspondingly, or, if there is a wall blocking the movement, it doesn't. This defines the body-environment system. The set of states is now *E* and has four elements corresponding to all the possible positions of the body. The transition function *A*:*E* × *M* → *E* tells where to move, and the rest is as above. The system E=(E,A,M,σ,S) is shown in Figure 3B. Let us now look at the agent. Suppose that it applies the following policy: (1) In the beginning move left; (2) if the previous move was to the left, then move right, otherwise move left. This can be modeled with a two-state automaton I=(I,S,B,μ,M) where *I* = {*L, R*}, *S* = {*s*_0_}, *B*(*L, s*_0_) = *R*, *B*(*R, s*_0_) = *L*, μ(*L*) = *l* and μ(*R*) = *r*. Now, the coupling ${\mathrm{LTS}}_{F}^{-1}\left(E\right)*{\mathrm{LTS}}_{P}^{-1}\left(I\right)$ is an automaton that realizes the policy in the environment, as shown in Figure 4A.

If the agent has a different embodiment in the same environment, then all of the automata will look different. Suppose that instead of the previous four actions, the agent has two: “rotate 90-degrees counterclockwise” (*C*),“forward one step” (*F*). Note that these are expressed in the local frame of the robot: It can either rotate relative to its current orientation, or it can move in the direction it is facing; the previous four actions were expressed as if in a global frame or the robot is incapable of rotation. Under the new embodiment, the unconstrained automaton with no sensor feedback is an infinite *binary tree*, with every node having two outgoing edges, labeled *C* and *F*, respectively, instead of the quaternary infinite tree depicted on Figure 3A. Instead of the four-state automaton of Figure 3, the automaton describing the environment transitions is a 16 state-automaton, because the orientation of the agent can now have four different values. See Figure 4B. Finally the automaton describing the internal mechanics of the agent I is a quasipolicy in these two actions, and finally, the coupling corresponds essentially to taking a path in the 16-state automaton above.

Note that there is a bisimulation between U and E which reflects the fact that from the point of view of an agent they are indistinguishable. This is natural because there is no sensory data, so from the agent's viewpoint it is unknowable whether or not it is embedded in an environment. A bisimulation is given as follows: Let *y*_0_ ∈ *Y* be the top-right corner and *x*_0_ ∈ *X* the root of the tree. Define *R* ⊆ *X* × *Y* be the minimal set satisfying the following conditions:

1. (*x*_0_, *y*_0_) ∈ *R*.
2. If (*x, y*) ∈ *R* and *m* ∈ *M*, then (*T*(*x, m*), *U*(*y, m*)) ∈ *R*.

**Example 3.2.** The 16-state automaton of Example 3.1 has four automorphisms corresponding to the rotation of the environment by 90 degrees counterclockwise. Each of those automorphisms corresponds to an auto-bisimulation. Mirroring is not an automorphism because the agent's rotating action fixes the orientation of the automaton.

**Figure 5:**
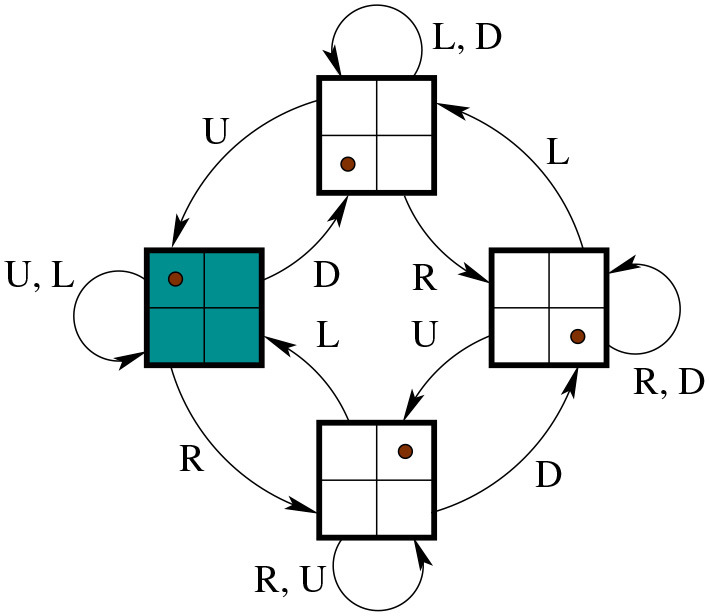
Consider the automaton E of Figure 3B from Example 3.1, but assume that the agent can “smell” a different scent in the top-left corner. This can be modeled by having a two-element set *S* = {0, 1} instead of a singleton, and *h*:*X* → {0, 1} such that *h*(*x*) = 0 iff *x* is not the top-left corner. The state with a scent is shaded.

**Example 3.3.** Figure 5 shows an example of how an automaton with non-trivial sensing could look. Jumping a little bit ahead, it will be seen that the labeling provided by *h* in this figure is not sufficient (a notion introduced in Definition 4.2).

## 4. Sufficient refinements and degree of insufficiency

This section presents the concept of *sufficiency*, which will be the main glue between enactivist philosophy and mathematical understanding of cognition. In Section 4.1 we introduce the main concepts and explain its profound relevance to enactivist modeling and how it can be a precursor to the emergence of meaning from meaningless sensorimotor interactions. In Section 4.2 we introduce the notion of minimal sufficient refinements, prove a uniqueness result about them, and show how they are connected to the classical notions of bisimulation as well as derived information state spaces^4^.

### 4.1. Sufficiency

The following consider the main definition of this work. It is based on the idea of sufficiency in LaValle (2006, Ch.11).

**Definition 4.1.** Let (*X, U, T*) be a transition system and *E* ⊆ *X* × *X* an equivalence relation. We say that *E* is *sufficient* or *completely sufficient*, if for all (*x, y*) ∈ *E* and all *u* ∈ *U*, if (*x, u, x*′) ∈ *T* and (*y, u, y*′) ∈ *T*, then (*x*′, *y*′) ∈ *E*.

This means that if an agent cannot distinguish between states *x* and *y*, then there are no actions it could apply to later distinguish between them. To put it differently, if the states are indistinguishable by an instant sensory reading, then they are in fact indistinguishable even through sensorimotor interaction. This is related to the equivalence relation known as Myhill-Nerode congruence in automata theory.

The equivalence relation of indistinguishability in the context of sensorimotor interactions is at its simplest the consequence of indistinguishability by sensors. Thus, we define sufficiency for labelings or sensor mappings:

**Definition 4.2.** A labeling *h*:*X* → *L* is called *sufficient* (or *completely sufficient*) iff for all *x, y, x*′, *y*′ ∈ *X* and all *u* ∈ *U*, the following implication holds:


$$\left(h\left(x\right)=h\left(y\right)\wedge \left(x,u,x^{\prime }\right)\in T\wedge \left(y,u,y^{\prime }\right)\in T\right)\Rightarrow h\left(x^{\prime }\right)=h\left(y^{\prime }\right).$$

**Proposition 4.3.** *If* (*X, U*, τ) *is an automaton, then h*:*X* → *L is sufficient if and only if for all x, y* ∈ *X and all u* ∈ *U*, *we have that if h*(*x*) = *h*(*y*), *then h*(τ(*x, u*)) = *h*(τ(*y, u*)).

*Proof*. Checking the definitions.               □

The above proposition is saying that when the sensorimotor system is deterministic, then sufficiency is equivalent to predictability.

There is a connection with the classical notion of bisimulation in classical transition systems theory (recall Definition 2.2):

**Proposition 4.4.** *An equivalence relation on a state space of an automaton* (*X, U*, τ) *is sufficient if and only if it is an autobisimulation*.

*Proof*. See Appendix B.               □

The above proposition can intuitively be interpreted as saying that a sufficient relation is one where different states with the same label are not only indistinguishable on their own, but are actually indistinguishable even by their consequences. Starting from one of two states with same labels, there is no way to ever find out which one of them it was, no matter how much will the agent investigate its environment, compare to the discussion in the end of Section 2.8.

Proposition 4.5 below is an important proposition on which the idea of derived I-spaces and combinatorial filters builds upon (LaValle, 2006, 2012; O'Kane and Shell, 2017), although as far as the authors are aware, in the literature, only the “if”-direction is mentioned. We say that a transition system (*X, U, T*) is *full*, if for all *x*_1_ ∈ *X* and all *u* ∈ *U* there exists at least one *x*_2_ ∈ *X* with (*x*_1_, *u, x*_2_).

**Proposition 4.5.** *Suppose* X=(X,U,T) *is a transition system. Let h*:*X* → *L be a labeling. Then* X/h *is an automaton if and only if* X *is full and h is sufficient*.

*Proof*. See Appendix B.               □

The above proposition brings together the ideas of a quotient, automaton and sufficiency. The idea of the quotient is that indistinguishable states can be in some circumstances considered the same and the idea of an automaton is that it is deterministic. The above proposition says that as far as the agent is concerned, if it equalizes indistinguishable states, then the world looks deterministic from the agent's perspective if and only if the underlying labeling satsifies Definition 4.2.

The sufficiency of an information mapping was introduced in LaValle (2006, Ch 11), and is encompassed by a sufficient labeling in this paper. In the prior context, it has meant that the current sensory perception together with the next action determine the next sensory perception. The elegance with respect to our principle (EA2) is that sufficiency is *not* saying that the agent's internal state corresponds to the environment's state (as is in representational models). Nor is it saying that the agent *predicts* the next action. It is saying, rather, that the agent's current sensation together with a choice of a motor command *determine* the agent's next sensation; and this statement is true only as a statement made about the system from outside, not as a statement which would reside “in the agent.” The sensation may carry no meaning at all “about” what is actually “out there.” However, if the agent has found a way to be coupled to the environment in a sufficient way, then sensations *begin* to be *about* future sensation. In this way meaning emerges from sensorimotor patterns. This relates to (EA3) and somewhat touches on the topic of perception (EA5). Furthermore, the property of determining future outcomes is related to (EA4) because that is what *skill* is. There is no potential to *reliably* engage with the environment in complex sensorimotor interactions, if the sensations do not *reliably* follow certain historical patterns.

Thus, the notion of sufficiency is considered by us to be of fundamental importance for enactivist-inspired mathematical modeling of cognition. The violation of sufficiency means that the current sensation-action pair does not correlate with the future sensation, making it harder to be attuned to the environment. Having a different sensation following the same pattern can be seen as a primitive notion of a “surprise.” This can be seen as aligning with the predictive coding and the free energy principle from neuroscience (Rao and Ballard, 1999; Friston and Kiebel, 2009; Friston, 2010), although our framework leaves the space to a clean non-representational interpretation while this is not obvious for these other frameworks. Does the notion of sufficient labelings capture the same ideas in a more general way? This is an open question for further research.

A generalization of sufficiency is *n*-sufficiency, in which the data of *n* previous steps is needed to determine the next label. Here, we define an *n*-chain.

**Definition 4.6.** An *n*-*chain* in X=(X,U,T) is a sequence


$$c=\left(x_{0},u_{0},\cdots ,x_{n-1},u_{n-1},x_{n}\right)\in {\left(X\times U\right)}^{n}\times X$$

such that $x_{i}\overset{u}{\to }x_{i+1}$ for all *i* < *n*. If *n* = 0, then by convention *c* = (*x*_*n*_). Let *E* ⊆ *X* × *X* be an equivalence relation. Let *k* < *n*. We say that two *n*-chains *c* = (*x*_0_, *u*_0_, …, *x*_*n*−1_, *u*_*n*−1_, *x*_*n*_), $c^{\prime }=\left(x_{0}^{\prime },u_{0}^{\prime },\ldots ,x_{n-1}^{\prime },u_{n-1}^{\prime },x_{n}^{\prime }\right)$ are (*T, E, k*)-equivalent if for all *i* < *k*, we have $u_{i}=u_{i}^{\prime }$ and $\left(x_{i},x_{i}^{\prime }\right)\in E$. An ∞-chain is defined in the same way as *n*-chain, except the sequences are infinite, without the “last” *x*_*n*_.

**Definition 4.7.** For a transition system X=(X,U,T), an equivalence relation *E* on *X* is called *n*-*sufficient* if there are no two (*T, E, n*)-equivalent *n*-chains


$$\begin{array}{l} c=\left(x_{0},u_{0},\ldots ,x_{n-1},u_{n-1},x_{n}\right)\text{ and }\\ c^{\prime }=\left(x_{0}^{\prime },u_{0}^{\prime },\ldots ,x_{n-1}^{\prime },u_{n-1}^{\prime },x_{n}^{\prime }\right) \end{array}$$

such that $\left(x_{n},x_{n}^{\prime }\right)\notin E$. A labeling *h*:*X* → *L* is called *n*-*sufficient* if *E*^*h*^ is *n*-sufficient (Recall Definition 2.34).

**Proposition 4.8.** *An equivalence relation E is* 0-*sufficient if and only if there is only one E-equivalence class, and a labeling function h is* 0-*sufficient if and only if it is constant*.

*Proof*. See Appendix B               □

**Proposition 4.9.** *An equivalence relation E* (*resp. a labeling h*) *is sufficient if and only if it is* 1-*sufficient*.

*Proof*. See Appendix B               □

**Proposition 4.10.** *Suppose n* < *m are natural numbers. If a labeling h is n-sufficient, then it is m-sufficient. The same holds for equivalence relations*.

*Proof*. See Appendix B               □

This enables us to define the degree of insufficiency:

**Definition 4.11.** The *degree of insufficiency* of the labeled automaton X=(X,U,τ,h,L) is defined to be the smallest *n* such that *h* is *n*-sufficient, if such *n* exists, and ∞ otherwise. Denote the degree of insufficiency of X by degins(X), or degins(*h*) if only the labeling needs to be specified and X is clear from the context.

The intuition is that the larger the degree of insufficiency of an environment X, the harder it is for an agent to be attuned to it. We talk more about the connection between attunement and sufficiency in the following sections.

### 4.2. Minimal sufficient refinements

In this section we prove that the minimal sufficient refinements are always unique (Theorem 4.19). This will follow from a deeper result that the sufficient equivalence relations form a complete sublattice of the lattice of all equivalence relations. This does not hold for *n*-sufficient equivalence relations for *n* > 1 (Example 4.20). We will then explore how the minimal sufficient refinements can be thought of as an enactive perceptual construct that emerges from the body-environment, brain-body, and brain-body-environment dynamics. The idea is that a minimal sufficient refiniment corresponds to an optimal attunement of the agent to the base labeling which corresponds to some minimal information that the agent is interested in the environment, such as death or life, danger or safety information. It is “optimal” by minimality and “attunement” by sufficiency. Our Theorem 4.19 states that such attunement is mathematically unique.

**Definition 4.12.** An equivalence relation *E* is a *refinement* of equivalence relation *E*′, if *E* ⊆ *E*′, also denoted $E^{\prime }\leq_{r}E$. A labeling function *h* is a refinement of a labeling function *h*′, if *E*^*h*^ is a refinement of *E*^*h*^′.

An important interpretation of the concept of a refinement is that a better sensor provides the agent with more information about the environment^5^. Each sensor mapping *h* induces a partition of *X* *via* its preimages, and refinement applies in the usual set-theoretic sense to the partitions when comparing sensors mappings. If a sensor mapping *h* is a refinement of *h*′, then it enables the agent to react in a more refined way to nuances in the environment. Using the partial ordering given by refinements, we obtain the *sensor lattice* (LaValle, 2019).

By a referee's request, let us give a couple of biological examples.

**Example 4.13** (First biological example). There is an accepted theory that primates see red color wavelength, because it enables them to distinguish ripe fruit from non-ripe. Assuming this theory is true, it is an example of a refinement which is to some extent “minimal” and to some extent “sufficient” (of course strictly speaking it is neither – in the same way as there is no ideal circle in the physical world). The minimality is seen in this example, because we perceive other things as red, even if it is completely unnecessary (certainly unnecessary to tell the ripeness of fruits). So we are not distinguishing “too much.” On the other hand, perceiving red color is a refinement of ripe/non-ripe which is only detected through stomach ache after the fruit has been already consumed. And it is sufficient in the sense that it is predictive of the original “base” labeling (ripe/non-ripe).

**Example 4.14** (Second biological example). Where our eyes look depends on the position of our head as well as the position of our eyes. Despite this, “looking up” (or “left,” “right” etc..) are not ambiguous, even though these can be achieved with virtually infinitely many different head-eye configurations. One way to understand how this invariance could emerge is through minimal sufficient refinements. Suppose at birth, every head-eye configuration is considered as a separate state, but we label them by what we see in any given (stable) situation. A minimal sufficient refinement of that labeling will never distinguish between different states in which the eyes are pointing in the same direction. So then, by learning the minimal sufficient refinements, the agent may learn eye-direction invariance.

### 4.3. Lattice of sufficient equivalence relations

Please refer to Appendix A in the Supplementary material for notations and definitions used in this section.

We will prove in this section that if (*X, U*, τ) is an automaton, the sufficient equivalence relations form a complete sublattice of (E(X),⊆). Given an automaton X=(X,U,τ), denote by $E_{\mathrm{suf}}^{U,\tau }\left(X\right)\subseteq E\left(X\right)$ the set of sufficient equivalence relations on *X*. When *U* and τ are clear from the context, we write just $E_{\mathrm{suf}}\left(X\right)=E_{\mathrm{suf}}^{U,\tau }\left(X\right)$.

**Theorem 4.15.** *Suppose* (*X, U*, τ) *is an automaton and suppose that* $E\subseteq E_{\mathrm{suf}}\left(X\right)$ *is a set of sufficient equivalence relations. Then* ∧E *and* ∨E *are sufficient. Thus*, $\left(E_{\mathrm{suf}}\left(X\right),\subseteq \right)$ *is a complete sublattice of* (E(X),⊆).

*Proof*. See Appendix B.               □

Suppose that a labeling *h* is very important for an agent. For example, *h* could be “death or life,” or it could be relevant for a robot's task. Suppose that *h* is not sufficient. The robot may want to find a sufficient refinement of *h*. Clearly a one-to-one *h*′ would do. However, assume that the agent has to use resources for distinguishing between states; thus, the fewer distinctions the better. This motivates the following definition. Recall Definition 4.12 of refinements.

**Definition 4.16.** Let (*X, U, T*) be a transition system and *E*_0_ ⊆ *X* × *X* an equivalence relation. *A minimal sufficient refinement* of *E*_0_ is a sufficient equivalence relation *E* which is a refinement of *E* such that there is no sufficient *E*′ with $E_{0}\leq_{r}E^{\prime }{<}_{r}E$.

Given a labeling *h*_0_ of a transition system (*X, U, T*), a *minimal sufficient refinement* of *h*_0_ is a labeling *h* such that *E*^*h*^ is a minimal sufficient refinement of $E^{h_{0}}$ (recall Definition 2.34).

**Example 4.17.** Let X=(X,U,τ) be an automaton where *X* = {0, 1}^*^, *U* = {0, 1} and τ(*x, b*) = *x*^⌢^*b* (concatenation of the binary string *x* with the bit *b*). Let *h*(*x*) = 1 if and only if the number of ones and the number of zeros in *x* are both prime; otherwise *h*(*x*) = 0. Then the only sufficient refinements of *h* are one-to-one.

**Example 4.18.** Let X be as above and let *h*:*X* → {0, 1} be such that if |*x*| is divisible by 3, then *h*(*x*) = 1; otherwise, *h*(*x*) = 0. Then *h* is not sufficient. Let *h*′:*x* ↦ {0, 1, 2} be such that


$$h^{\prime }\left(x\right)\equiv |x|\mathrm{mod}3.$$

Then *h*′ is a minimal sufficient refinement of σ.

**Theorem 4.19.** *Consider an automaton* X=(X,U,τ) and let *E*_0_ *be an equivalence relation on X. Then a minimal sufficient refinement of E*_0_ *exists and is unique*.

*Proof*. See Appendix B               □

Theorem 4.19 fails, if “automaton” is replaced by “transition system,” or if “sufficient” is replaced by “*n*-sufficient” for *n* > 1 (recall Definition 4.7)

**Example 4.20** (Failure of uniqueness for *n*-sufficiency). Let *X* = {0, 1, 2, 3, 4, 5}, *U* = {*u*_0_} and


$$\tau \left(0,u_{0}\right)=1,\tau \left(1,u_{0}\right)=2,\tau \left(2,u_{0}\right)=2,$$

and


$$\tau \left(3,u_{0}\right)=4,\tau \left(4,u_{0}\right)=5,\tau \left(5,u_{0}\right)=5.$$

Let *E*_0_ be an equivalence relation on *X* such that the equivalence classes are {0, 1, 3, 4}, {2} and {5}. Then this relation is not 2-sufficient, because (0, *u*_0_, 1, *u*_0_, 2) and (3, *u*_0_, 4, *u*_0_, 5) are (*T, E*_0_, 2)-equivalent, but 2 and 5 are not *E*_0_-equivalent. Let *E*_1_, *E*_2_ ⊆ *E*_0_ be equivalence relations with equivalence classes as follows:


$$E_{1}:\left\{0,1\right\},\left\{3,4\right\},\left\{2\right\},\left\{5\right\},$$

$$E_{2}:\left\{0,4\right\},\left\{1,3\right\},\left\{2\right\},\left\{5\right\}.$$

Then *E*_1_ and *E*_2_ are refinements of *E*_0_. They are both 2-sufficient, because there doesn't exist any (*T, E*_1_, 1) or (*T, E*_2_, 1) equivalent 2-chains. They are also both ≤_*r*_-minimal with this property which can be seen from the fact that they are actually ≤_*r*_-minimal refinements of *E*_0_ as equivalence relations (not only as sufficient ones).

**Example 4.21** (Failure of uniqueness for transition systems). Let *X* = {0, 1, 2, 3, 4}, *U* = {*u*_0_} and *T* = {(0, *u*_0_, 3), (2, *u*_0_), 4}. Let *E*_0_ be the equivalence relation with the equivalence classes {0, 1, 2}, {3} and {4}. Then *E*_0_ is not sufficient, because (0, 2) ∈ *E*_0_, but (3, 4) ∉ *E*_0_. Let *E*_1_ and *E*_2_ be the refinements of *E*_0_ with the following equivalence classes:


$$E_{1}:\left\{0,1\right\},\left\{2\right\},\left\{3\right\},\left\{4\right\},$$

$$E_{2}:\left\{0\right\},\left\{1,2\right\},\left\{3\right\},\left\{4\right\}.$$

Now it is easy to see that both *E*_1_ and *E*_2_ are sufficient refinements of *E*_0_, and by a similar argument as in Example 4.20 they are both minimal. The reason why this is possible is the odd behavior of the state 2 which doesn't have out-going connections. Such odd states are the reason why the decision problem “Does there exist a sufficient refinement with *k* equivalence classes?” is NP-complete for finite transition systems (O'Kane and Shell, 2017).

*Remark*. It is worth noting that Theorems 4.15 and 4.19 do not assume anything about the cardinality of *X* or of *U*, other structure on them (such as metric or topology) nor anything about the function τ or the relation *E*_0_. Keeping in mind potential applications in robotics, *X* and *U* could be, for instance, topological manifolds, and τ a continuous function, or *X* could be a closed subset of ℝ^*n*^, *U* discrete and τ a measurable function, or any other combination of those. In each of those cases, the sublattice of sufficient equivalence relations is complete, as per Theorem 4.15, and every equivalence relation *E*_0_ on *X* admits a unique minimal sufficient refinement as per Theorem 4.19.

Recall Definition 2.10 of an equivalence relation preserving function. We say that an equivalence relation *E* on *X* is *closed under* *f*:*X* → *X* if for all *x* ∈ *X*, we have (*x, f*(*x*)) ∈ *E*. If *E* is closed under *f*, then *f* is *E*-preserving: given (*x, x*′) ∈ *E*, we have (*x, f*(*x*)), (*x*′, *f*(*x*′)) ∈ *E*, because *E* is closed under *f*. Now by transitivity of *E* we have (*f*(*x*), *f*(*x*′)) ∈ *E*, so *f* is *E*-preserving.

**Definition 4.22.** Let *f*:*X* → *X* be a bijection. The induced *orbit equivalence relation* is the relation *E*_*f*_ on *X* defined by $\left(x,x^{\prime }\right)\in E_{f}\Leftrightarrow \left(\exists n\in \mathbb{Z}\right)\left(f^{n}\left(x\right)=x^{\prime }\right)$, in which *f*^*n*^(*x*) is defined by induction as: *f*^0^(*x*) = *x*, *f*^*n*+1^(*x*) = *f*(*f*^*n*^(*x*)), *f*^*n*−1^(*x*) = *f*^−1^(*f*^*n*^(*x*)).

**Theorem 4.23.** *If f is an automorphism of the automaton* (*X, U*, τ), *then E*_*f*_ *is a sufficient equivalence relation*.

*Proof*. See Appendix B               □

**Theorem 4.24.** *Let* X=(X,U,τ) *be an automaton and E be an equivalence relation on X. Suppose f*:*X* → *X is an automorphism such that E is closed under f. Let E′ is the minimal sufficient refinement of E. Then E′ is closed under f and* $E\leq_{r}E^{\prime }\leq_{r}E_{f}$.

*Proof*. See Appendix B               □

**Figure 6:**
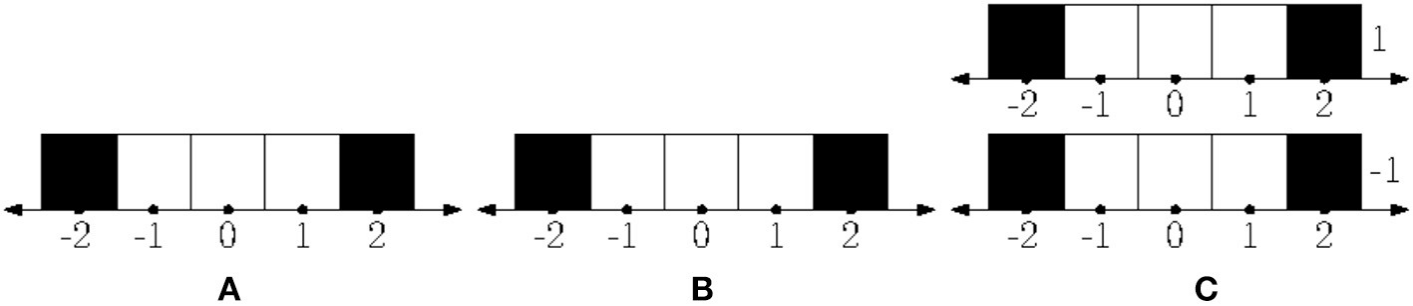
**(A)** One-dimensional lattice environment described in Example 4.25. **(B)** State space of the agent 0. **(C)** State space of the agent 1. The states for which the value of the sensor mapping is 0 are shown in black.

**Example 4.25.** Consider the environment which is a one-dimensional lattice of length five, *E* = {−2, −1, 0, 1, 2}, in which the corners “smell bad”; thus, we have a sensor mapping *h*:*E* → *S*, *S* = {0, 1} defined by *h*(*n*) = 0 ⇔ |*n*| = 2; see Figure 6A. Consider two agents in this environment. Both are equipped with the same *h* sensor, but their action repertoires differ. Both have two possible actions. One has actions *L*= “move left one space” and *R*= “move right one space,” and the other one has actions *T*= “turn 180 degrees” and *F*= “go forward one space.” Let *M*_0_ = {*L, R*} and *M*_1_ = {*T, F*}. Thus, these agents have a slight difference in embodiment. Although both of them can move to every square of the lattice in a very similar way (almost indistinguishable from the outside perspective), we will see that the differences in embodiment will be reflected in that the minimal sufficient refinements will produce non-equivalent “categorizations” of the environment. The structures that emerge from these two embodiments will be different. These agents *enact* different environments, although physically the environments are the same, as congruent with tenet (EA3).

First, we define the SM-systems that model these agents' embodiments in *E*. The first agent does not have orientation. It can be in one of the five states, and the state space is *X*_0_ = *E* (Figure 6B). For the second agent, the effect of the *F* action depends on the orientation of the agent (pointing left or pointing right). Thus, there are ten different states the agent can be in, yielding *X*_1_ = *E* × {−1, 1} (Figure 6C). The effects of motor outputs are specified completely (*L* means moving left, and so on), whereas the agent's internal mechanisms are left completely open, so our systems will be quasifilters. According to Remark 2.18, we can work with a labeled automaton instead. Hence, let τ_0_:*X*_0_ × *M*_0_ → *X*_0_ be defined by τ_0_(*x, L*) = max(*x* − 1, −2) and τ_0_(*x, R*) = min(*x* + 1, 2). For the other agent, let τ_1_((*x, b*), *T*) = (*x*, −*b*) and τ_1_((*x, b*), *F*) = (min(max(*x* + *b*, −2), 2), *b*). Now we have labeled automata $X_{0}=\left(X_{0},M_{0},\tau_{0},h,S\right)$ and $X_{1}=\left(X_{1},M_{1},\tau_{1},h,S\right)$.

**Figure 7:**
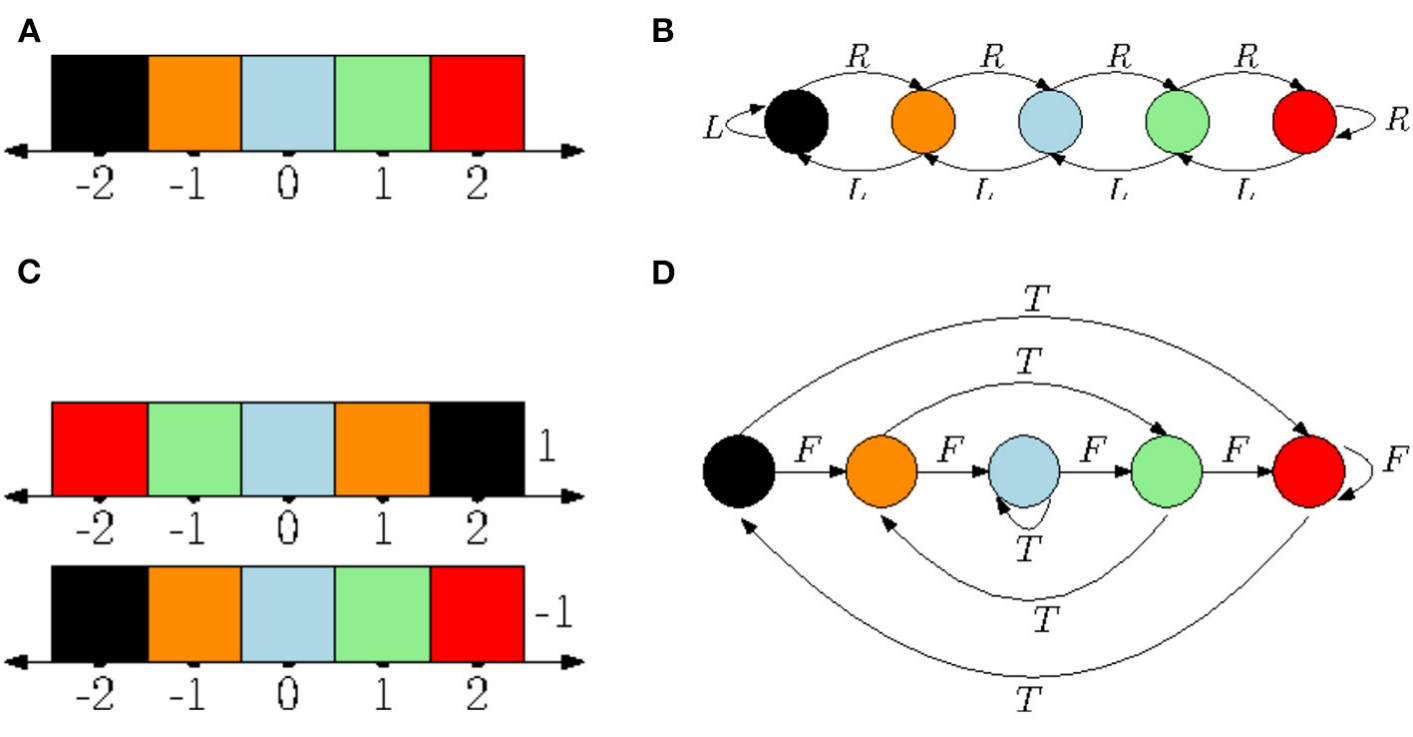
**(A)** State space of the agent 0 categorized by *h*_0_, states that belong to the same class are colored with the same color. **(B)** Resulting quotient $X_{0}/h_{0}$ for the agent 0. **(C)** State space of the agent 1 categorized by *h*_1_, states that belong to the same class are colored with the same color. **(D)** Resulting quotient $X_{1}/h_{1}$ for the agent 1.

It is not hard to see that the one-to-one map *h*_0_:*X*_0_ → {−2, −1, 0, 1, 2} with *h*_0_(*x*) = *x* is a sufficient refinement of *h* which is minimal (see Figure 7A). Thus, every state needs to be distinguished by the agent for it to be possible to determine the following sensation from the current one. The derived information space automaton $X_{0}/h_{0}$ isomorphic to $X_{0}$ (Proposition 2.36).

For the second automaton, consider the labeling *h*_1_:*X*_1_ → {−2, −1, 0, 1, 2} defined by *h*_1_(*x, b*) = *b*·*x* (see Figure 7C).

**Claim**. *h*_1_ is a minimal sufficient refinement of *h* in $X_{1}$.

*Proof*. See Appendix B               □

Both minimal sufficient labelings, *h*_0_ and *h*_1_ have five values; thus, they categorize the environment into five distinct state-types. However, the resulting derived information spaces are different in the sense that the quotients $X_{0}/h_{0}$ and $X_{1}/h_{1}$ are not isomorphic; compare Figure 7B with Figure 7D.

**Figure 8:**
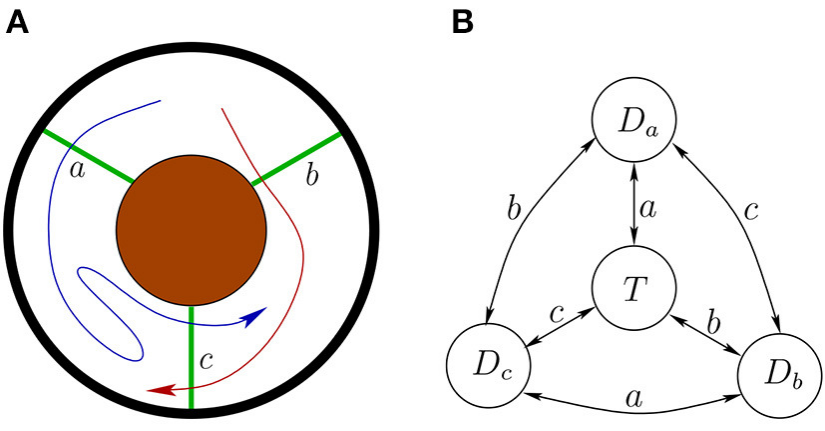
**(A)** Two point-sized independent bodies move along continuous paths in an annulus-shaped region in the plane. There are three sensor beams, *a*, *b*, and *c*. When each is crossed by a body, its corresponding symbol is observed. Based on receiving a string of observations, the task is to determine whether the two bodies are together in the same region, with no beam separating them. **(B)** The minimal filter as a transition system has only 4 states: *T* means that they are together, and *D*_*x*_ means that are in different regions but beam *x* separates them. Each transition is triggered by the observation when a body crosses a beam.

**Example 4.26.** Figure 8A shows a filtering example from Tovar et al. (2014). More complex versions have been studied more recently in O'Kane and Shell (2017), and are found through automaton minimization algorithms and some extensions. It can be shown that this example's four-state derived information space depicted on Figure 8B corresponds to the unique minimal sufficient refinement of the labeling that only distinguishes between “are in the same region” and “are not in the same region.” To see this, first note that this labeling is sufficient (since it can be represented as an automaton, this follows from Theorem 4.5). It follows from Theorem 4.19 that if this labeling is not minimal, then there is a minimal one which is strictly coarser, and so can be obtained by merging the states in the automaton of Figure 8B. This is impossible: the state *T* cannot be merged with anything because it violates the base-labeling; if, say *D*_*a*_ and *D*_*c*_, are merged, then transition *a* will lead to inconsistency as it can lead either to *D*_*b*_ (from *D*_*c*_) or to *T* (from *D*_*a*_). This proves that this derived information space is indeed minimal sufficient, and by Corollary 4.19 there are no others up to isomorphism.

### 4.4. Computing sufficient refinements

This section sketches some computational problems and presents computed examples. The problem of computing the minimal sufficient refinement in some cases reduces to classical deterministic finite automaton (DFA) minimization, and in other cases it becomes NP-hard (O'Kane and Shell, 2017).

Consider an automaton (*X, M*, τ) and a labeling function *h*_0_, and the corresponding labeled automaton described using the quintuple (*X, M*, τ, *h*_0_, *L*). Suppose that the automaton (*X, M*, τ) corresponds to that of an body-environment system. Hence, *X* corresponds to the states of this coupled system. Suppose *h*_0_ is not sufficient and consider the problem of computing a (minimal) sufficient refinement of *h*_0_, that is, the coarsest refinement of *h*_0_ that is sufficient.

Despite the uniqueness of the minimal sufficient refinement of *h*_0_ (by Corollary 4.19), we can argue that the formulation of the problem, in particular, the input, can differ based on the level at which we are addressing the problem (for example, global perspective, agent perspective or something in between). Since the labeled automaton corresponding to an agent-environment coupling is described from a global perspective, the input to an algorithm that addresses the problem from this perspective is the labeled automaton $A=\left(X,\tau ,M,h_{0},L\right)$ itself. Then, the problem is defined as given A compute $A^{\prime }=\left(X,M,\tau ,h,L\right)$ such that *h* is the minimal sufficient refinement of *h*_0_.

A special case of this problem from the global perspective occurs if the preimages of *h*_0_ partition *X* in two classes which can be interpreted as the “accept” and “reject” states, for example, goal states at which the agent accomplishes a task and others. Furthermore, suppose that the initial state of the agent is known to be some *x*_0_ ∈ *X*. Then, computing a minimal sufficient refinement becomes identical to minimization of a finite automaton, that is, given a DFA (*X, M*, τ, *x*_0_, *F*) in which *x*_0_ is the initial state and *F* is the set of accept states find $\left(X^{\prime },M,\tau^{\prime },x_{0}^{\prime },F^{\prime }\right)$ such that no DFA with fewer states recognizes the same language. Existing algorithms, for example Hopcroft (1971), can be used to compute a minimal automaton.

Here, we also consider this problem from the agent's perspective for which the information about the environment states is obtained through its sensors, more generally, through a labeling function. Note that by agent's perspective we do not necessarily imply that the agent is the one making the computation (or any computation) but it means that no further information can be gathered regarding the environment other than the actions taken and what is sensed by the agent. At this level we address the following problem; given a set *M* of actions, a domain *X*, and a labeling function *h*_0_ defined on *X*, compute the minimal sufficient refinement of *h*_0_. The crux of the problem is that unlike the global perspective described above, the labeled automaton A is not given, in particular, the state transitions are not known a priory. Instead, the information regarding the state transitions can only be obtained locally by means of applying actions and observing the outcomes, that is, through sensorimotor interactions. Hence, the current body-environment state is also not observable. To show that an algorithm exists to compute a sufficient refinement of *h*_0_ at this level, we propose an iterative algorithm (Algorithm 1) that explores *X* through agent's actions and sensations by keeping the history information state, that is, the history of actions and sensations (labels). We then show, by empirical results, that the sufficient refinement computed by Algorithm 1 is minimal for the selected problem.

**Algorithm 1:** 

1:	**Input:** *h*_0_, *l*_0_, *M*
2:	**Initialize:** *H* ← ∅, *h* ← *h*_0_, *s* ← *s*_0_
3:	**for** each step **do**
4:	*m* ← policy(*s*)
5:	apply action *m* and obtain resulting *s*′
6:	add (*s, m, s*′) to *H*
7:	**if** ∃(*s, m, s*″) ∈ *H* such that *s*′ ≠ *s*″ **then**
8:	*h* ← split(*h, s*)
9:	**if** *there are labels that can be merged* **then**
10:	*h* ← *merge*(*h, H, h*_0_)
11:	*s* ← *s*′

The functioning of Algorithm 1 is as follows. Starting from an initial sensation *s*_0_ = *h*(*x*_0_), the agent moves by taking an action^6^ given by the mapping policy:*L* → *M*. Particularly, we used a fixed policy which samples an action *m* from a uniform distribution over *M* for each *s* ∈ *S*. In principle, any policy that ensures all states that are reachable from *x*_0_ will be visited infinitely often should be enough. The history information state is implemented as a list, denoted by *H*, of triples (*s, m, s*′) such that *s* = *h*(*x*) and *s*′ = *h*(*x*′) in which *x*′ = τ(*x, m*). At each step, it is checked whether the current sensation is consistent with the history (Line 7). Current sensation is inconsistent with the history if there exists a triple (*s, m, s*″) in the history such that *s*′ ≠ *s*″. If it is not consistent then the label is split, which means that *h*^−1^(*s*) is partitioned into two parts *P* and *Q*. In particular, we apply a balanced random partitioning, that is, we select *P* and *Q* randomly from a uniform distribution over the partitions of *h*^−1^(*s*) that have two elements with balanced cardinalities. The labeling function is updated by a split operation as


$$h\left(x\right):=\{\begin{array}{ll} s_{Q} & \text{if }x=Q\\ s_{P} & \text{if }x=P\\ h\left(x\right) & \text{otherwise}. \end{array}$$

Recall that labels or subscripts do not carry any meaning from the agent's perspective.

Even a trivial strategy that splits the preimage of the label seen at each step would succeed computing a sufficient refinement. However, this would result in *h* being a one-to-one mapping. Hence, the finest possible refinement. Splitting only at the instances when an inconsistency is detected might reach a coarser refinement that is sufficient but there might be more equivalence classes than the ones induced by the minimal sufficient refinement of *h*_0_. Therefore, a merge operation is introduced (Line 10). Let *s* and *s*′ be two distinct labels for which $\exists s^{\prime \prime }\in h_{0}\left[X\right]$ such that $h^{-1}\left(s\right)\subseteq h_{0}^{-1}\left(s^{\prime \prime }\right)$ and $h^{-1}\left(s^{\prime }\right)\subseteq h_{0}^{-1}\left(s^{\prime \prime }\right)$. Let *t* denote a triple in *H* and let *t*_*k*_, *k* = 1, 2, 3, denote the *k*^*th*^ element of that triple. Suppose *s*′ = *s*, if there are at least *N* number of triples in *H* such that for each triple *t*, (*t*_1_, *t*_2_) = (*s, m*) and ∀*m* ∈ *M* and ∀*t, t*′ ∈ *H* such that $\left(t_{1},t_{2}\right)=\left(t_{1}^{\prime },t_{2}^{\prime }\right)=\left(s,m\right)$ it is true that $t_{3}=t_{3}^{\prime }$ then labels *s* and *s*′ are merged. The merge procedure goes through all labels and updates *h* as


$$h\left(x\right):=\{\begin{array}{ll} s & \text{if }h\left(x\right)\in \left\{s,s^{\prime }\right\}\\ h\left(x\right) & \text{otherwise.} \end{array}$$

for each pair of labels *s* and *s*′ that satisfies the aforementioned condition. Note that in principle, one can merge two labels regardless of the number of occurrences in the history. However, we noticed that this can result in oscillatory behaviour between split and merge operations especially for states that are reached less frequently. At present, we considered *N* as a tunable parameter and we know that it depends on the cardinality of the state space *X* such that larger the number of states, larger *N* should be. The problem of defining *N* as a function of the problem description remains open.

**Figure 9:**
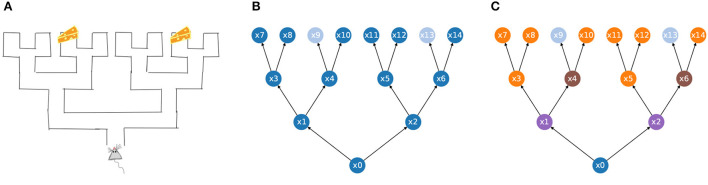
**(A)** Cheese maze defined in Example 4.27 **(B)** Labeled automaton with initial labeling *h*_0_ corresponding to the cheese-maze example. **(C)** Minimal sufficient refinement of *h*_0_. Self-loops at the leaf nodes are not shown in the figure.

In the following, we present an illustrative example to show the practical implications of the previously introduced concepts in Section 4.2. In particular, we show how a simple algorithm like Algorithm 1 can be used by a computing unit which relies only on the sensorimotor interactions of an agent to further categorize the environment such that there are no inconsistencies in terms of the actions taken by the agent and the resulting sensations with respect to an initial categorization induced by *h*_0_ (Figure 9C).

**Figure 10:**
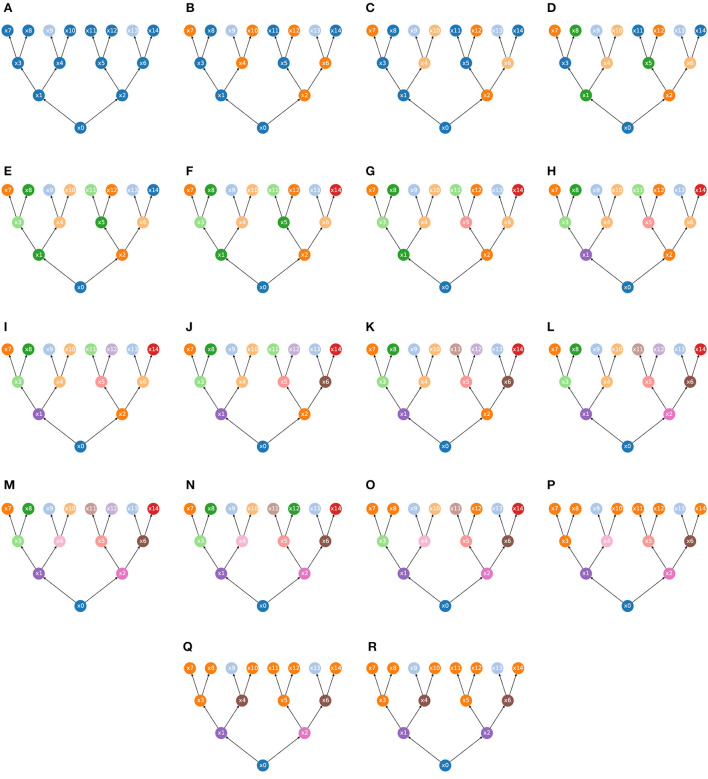
**(A)** Labeled automaton with labefigure/ling function *h* = *h*_0_; same colored states belong to the figure/same equivalence class. **(B–Q)** Updating *h* by Algorithm 1 through splitting and merging of the labels. **(R)** Labeled automaton with the labeling function *h* that is the minimal sufficient refinement of *h*_0_.

**Example 4.27.** Consider an agent (a mouse) that is placed in a maze where certain paths lead to cheese and others do not (see Figure 9A). At each intersection the agent can go either left or right and it can not go back. Hence, at each step the agent takes one of the two actions; go right or go left. Figure 9B shows the corresponding automaton with 15 states describing the agent-environment system together with the initial labeling *h*_0_ that partitions the state space into states in which the agent has reached a cheese (light blue) and others (dark blue). The initial state *x*_0_ is when the agent is at the entrance of the maze. Once the end of the maze is reached (a leaf node) the state does not change regardless of which action is taken. After a predetermined number of steps the system reverts back to the initial state, similar to an episode in the reinforcement learning terminology (see, for example, Sutton and Barto, 2018). However, despite the system going back to the initial state the history information state still includes the prior actions and sensations. Figure 10 reports the updates of *h*, initialized at *h*_0_, by Algorithm 1 being run for 1,000 steps. It converged to a final labeling *h* (Figure 10R), that is the minimal sufficient refinement of *h*_0_, in 435 steps. For 20 initializations of Algorithm 1 for the same problem, on average, it took 364 steps to converge to a minimal sufficient refinement of *h*_0_ (Figure 9C).

We have also applied the same algorithm to variations of this example with different depths of maze and different number of cheese and cheese placements (varying *h*_0_). Empirical evidence shows that the same algorithm was capable of consistently finding the minimal sufficient refinement of the initial labeling. However, it is likely that it might fail for more complicated problems, for example, when the number of actions are significantly larger. It remains an open problem finding a provably correct algorithm for computing the minimal sufficient refinement of *h*_0_ from the agent's perspective.

### 4.5. Sufficiency for coupled SM-systems

Section 2 introduced SM-systems, including the special class of quasifilters. We showed that quasifilters can be thought of as labeled transition systems, and we worked with such systems in Sections 4, 4.4. Let us see how do the concepts introduced in those sections work for SM-systems. We also defined *coupling* of SM-systems (Definition 2.22), but we have not defined what it means for a coupling to be “good.” We will use sufficiency to approach this subject.

Let E=(E,(S×M),T) and I=(I,S×M,B) be SM-systems. We think intuitively of E as “the environment” and I as the “agent,” even though they share the set of sensorimotor parameters *S* × *M*. When is the coupling E*I “successful”? Given another $I^{\prime }=\left(I^{\prime },S\times M,B^{\prime }\right)$, how can we compare I and $I^{\prime }$ in the context of E? The coupled system E*I is not labeled; therefore, we cannot apply the definition of sufficiency. However, as soon as we apply some labeling to it, we can. There are many different ways to do it, intuitively corresponding to the “agent's perspective,” the “environment's perspective” and a “god's perspective” (or “global perpsective”).

The first one is the labeling *h*:*E* × *I* → *I*, which is the projection to the right coordinate, *h*_*I*_(*e, i*) = *i*. The second one is the projection to the left coordinate *h*_*E*_(*e, i*) = *i*, and the third one is the labeling of states by themselves, *h*_*G*_(*e, i*) = (*e, i*). Clearly, *h*_*G*_ is a refinement of both *h*_*E*_ and *h*_*I*_. Yet another option is to use the sensory data as labelings, which is a coarser labeling than *h*_*I*_. Or perhaps there was already a labeling *h*:*E* → *S* to begin with, so then we can ask about the property of ĥ:*E* × *I* → *S* defined by ĥ(*e, i*) = *h*(*e*). We focus on what we called the agent's perspective, *h*_*I*_, for the rest of this section.

Recall Definition 4.11 of the degree of insufficiency. Given SM-systems E (environment) and I (agent), we can ask what is the degree of insufficiency of *h*_*I*_ in E*I? The smaller the degree, the better the agent is attuned to the environment. This says something about the way in which the agent is adapted or attuned to the environment without attributing contentful states or representations to the agent in alignment with (EA2) and (EA4).

Let E, I, and $I^{\prime }$ be SM-systems. When is $\mathrm{degins}\left(E*I,h_{I}\right)<\mathrm{degins}\left(E*I^{\prime },h_{I^{\prime }}\right)$? Of course, if I is fully constrained (Definition 2.31), then degins(E*I)=∞. This corresponds to the agent never engaging in any sensorimotor interaction with the environment. No wonder that it can always “predict” the result of such passive existence. Assume, however, that there some constraints on the coupling. For example, we may demand that the agent must regularly visit states of some particular type to survive. Subject to such constrains, what can we say about degins(E*I)? This seems to be a good preliminary notion^7^ of attunement.

## 5. Discussion

In the introduction we defined our basic enactivist tenets:

(EA1) Embodiment and the inseparability of the brain-body-environment system,
(EA2) Grounding in sensorimotor interaction patterns, not in contentful representations.
(EA3) Emergence from embodiment, enactment of the world,
(EA4) Attunement, adaptation, and skill as possibilities to reliably engage in complicated patterns of activity with the environment.
(EA5) Perception as sensorimotor skills.

We developed a model of sensorimotor systems and coupling for which the purpose is to account for cognition mathematically, but in congruence with the principles (EA1)–(EA5). The principle (EA1) is intrinsic in the ways SM-systems are supposed to model brain-body and body-environment dynamics. The central ingredient is the control set *S* × *M* in all of those systems which include “motor” and “sensory” part; it is *impossible* in our framework to model say the environment without acknowledging the way in which the body is *part of* it. The approach that the actions of an agent depend solely on the history of its sensorimotor interactions with the environment, our approach is well in the scope of (EA2). We do not assume any representational or symbolic content possessed by the SM-systems. We do not evaluate them normatively by the “correctness” of their internal states, but rather by the ways in which they are, or can be, coupled to the environment and whether their sensory apparatus generates a sufficient sensor mapping or not. Coupling of SM-systems is defined so that two systems constrain each other. Thus, when an agent is coupled to the environment, they constrain each other, thereby creating new global properties of the body-environment system.

The principle (EA4) is mostly discussed in connection with minimal sufficient refinements. Given a labeling, or a categorization, or an equivalence relation on the state space, one can ask how well does this labeling “predict itself.” The interpretation of this labeling can be anything from a sensor mapping to the labeling of environmental states by the internal states of the agent which coincide with them (this is not representation, this is mere co-occurence; see enactivist interpretation of the place cells in Hutto and Myin (2017) for comparison). A sufficient sensor mapping can be achieved in many different ways. In Section 4.4 we present a way in which the agent “develops” new sensors to be better attuned to the environment and in that way finds a sufficient sensor mapping. Another way for the agent would be to learn to act in a way that excludes “unpredictability.” Both are examples of situations where the agent “structures” its own body-environment reality and gains skill. Finally, perception (EA5) can be understood as sensorimotor patterns on a microlevel. On the other hand, the agent engage in a sensorimotor activity locally without making big moves, such as moving the eyes without moving the body. The result of such sensorimotor interaction is another labeling function on a macro level.

In this paper, we not only presented mathematical definitions, but proved a number of propositions and theorems about them. There would be (and we hope there will be!) much more of them, but they did not fit in this expository work for which the main purpose was to demonstrate the connection of the mathematics in question with the enactive philosophy of mind.

We have already developed more concepts and theorems on top of this framework, including notions of *degree of insufficiency, universal covers, hierarchies*, and *strategic sufficiency*, but these are omitted here due to space limitations.

In other, more mathematical work, we plan to concentrate on working out mathematical and logical details of the proposed theory as well as applying the ideas to fundamental questions in robotics and autonomous systems, control theory, machine learning, and artificial intelligence.

## Data availability statement

The original contributions presented in the study are included in the article/Supplementary material, further inquiries can be directed to the corresponding authors.

## Author contributions

VW, BS, and SL developed the mathematical theory together over the past 2 years after extensive collaborative sessions. The primary author is VW, who wrote the most among the authors. VW contributed more to mathematical proofs. BS contributed more to computation. In addition to individual contributions, SL also played a supervisory role. All authors contributed to writing.

## Funding

This work was supported by a European Research Council Advanced Grant (ERC AdG, ILLUSIVE: Foundations of Perception Engineering, 101020977), Academy of Finland (projects PERCEPT 322637, CHiMP 342556), and Business Finland (project HUMOR 3656/31/2019). All authors are with the Center for Ubiquitous Computing, Faculty of Information Technology and Electrical Engineering, University of Oulu, Finland.

## Conflict of interest

The authors declare that the research was conducted in the absence of any commercial or financial relationships that could be construed as a potential conflict of interest.

## Publisher's note

All claims expressed in this article are solely those of the authors and do not necessarily represent those of their affiliated organizations, or those of the publisher, the editors and the reviewers. Any product that may be evaluated in this article, or claim that may be made by its manufacturer, is not guaranteed or endorsed by the publisher.

## Footnotes

^1^This idea of a reachable set G is the simplest way to formalize affordances.

^2^This means a full specification of the environment, the agent's body, its brain, their coupling, as well as the initial states.

^3^We do not mean to say that no data is always the same as some other data. We are talking here about an agent that *never* receives any data, or an agent that *always* receives the same data. Thus, it cannot rely on any “change” between having and not having any sensory input. Thus, there is no “presense in absence” paradox here.

^4^There could be an interesting relationship between this concept and the free energy principle proposed by K. Friston. A system which is attuned to its environment in a sufficient way can be interpreted by an inspector as a system that is making ] predictions about its environment.

^5^Here we are not talking about contentful or semantic information, but merely about correlational information in the philosophical sense.

^6^This can either be in a real environment or in a realistic simulation.

^7^Further research will indicate how much of this will be accepted by the most radical enactivists.
