# Supplementary material for: An enactivist-inspired mathematical model of cognition
Source: Front Neurorobot. 2022 Sep 30;16:846982. doi: 10.3389/fnbot.2022.846982 (PMC9561910; doi:10.3389/fnbot.2022.846982)
Supplement: Supplementary file 1 [file Data_Sheet_1.pdf]

Supplementary Material to “Enactivist-inspired Mathematical Model of Cognition” by Vadim Weinstein, Basak Sakcak, and Steven M. LaValle

A APPENDIX: LATTICES OF EQUIVALENCE RELATIONS

Let $(L, \leqslant)$ be an ordered set. An upper bound of a subset L' is an element u such that $l \leqslant u$ for all $l \in L$. It is a least upper bound, it is an upper bound and $u \leqslant x$ for any other upper bound x of L'. If the least upper bound exists, then it is unique, for if u and u' are least upper bounds, then by definition we have $u \leqslant u'$ and $u' \leqslant u$. Symmetrically one defines the lower bound and the greatest lower bound which also turns out to be unique. In lattice theory, the least upper bound is often called join and the greatest lower bound is called meet.

Definition A.1. A lattice is an ordered set $(L, \leqslant)$ such that for any two elements $a, b \in L$ there is a join $j = a \vee b$ and a meet $m = a \wedge b$. We may denote a lattice as a quadruple $(L, \leqslant, \wedge, \vee)$ where $\wedge$ and $\vee$ are the join and meet operations.

By induction one can show that in a lattice every finite subset has a join as well as a meet. A lattice is called complete, if this extends to all, not necessarily finite, subsets:

Definition A.2. A lattice $(L, \leqslant)$ is complete, if for all $L' \subseteq L$ there are a join $j = \bigvee L'$ and a meet $m = \bigwedge L'$.

A lattice $(L, \leqslant, \wedge, \vee)$ is a sublattice of $(L', \leqslant', \wedge', \vee')$, if $L \subseteq L'$, for all $l_0, l_1 \in L$ we have

$$l_0 \leqslant l_1 \iff l_0 \leqslant' l_1,$$

and for all $L_0 \subseteq L$ we have that if $\bigwedge L_0$ exists, then $\bigwedge L_0 = \bigwedge' L_0$ and if $\bigvee L_0$ exists, then $\bigvee L_0 = \bigvee' L_0$. It is a complete sublattice, if $(L, \leqslant)$ is a complete lattice.

Given a set non-empty $A \subseteq X^2$, the equivalence relation generated by A, denoted $\langle A \rangle$, is the smallest equivalence relation on X which contains all pairs that are in A, so

$$\langle A \rangle = \bigcap \{E \supset A \mid E \text{ is an equivalence relation on } X\},$$

or equivalently, $(x, x') \in \langle A \rangle$, iff there exist $x_1, \ldots, x_n$ such that the pairs

$$(x, x_1), (x_1, x_2), \ldots, (x_{n-1}, x_n), (x_n, x')$$

are all in $A \cup A^T$ where $A^T = \{(x', x) \mid (x, x') \in A\}$.

Lemma A.3. If E is any equivalence relation such that $A \subseteq E$, then $\langle A \rangle \subseteq E$.

Proof. Suppose $(x, x') \in \langle A \rangle$. Then there is a sequence of pairs

$$(x, x_1), (x_1, x_2), \ldots, (x_{n-1}, x_n), (x_n, x')$$

that are all in $A \cup A^T$. Since $A \subseteq E$, also $A^T \subseteq E$ and so $A \cup A^T \subseteq E$, from which we see that all these pairs are in E. By transitivity of E, $(x, x') \in E$.

Definition A.4. Let X be any set and let $\mathcal{E}(X)$ be the set of all equivalence relations on X. Then $(\mathcal{E}(X), \subseteq)$ is an ordered set. Given a set of equivalence relations $\mathcal{E} \subseteq \mathcal{E}(X)$, define

$$\bigwedge \mathcal{E} = \bigcap \mathcal{E} = \{(x_1, x_2) \in X^2 \mid (\forall E \in \mathcal{E})((x_1, x_2) \in E)\}$$

Then $\bigwedge \mathcal{E}$ is an equivalence relation such that $\bigwedge \mathcal{E} \subseteq E$ for all $E \in \mathcal{E}$, so it is a lower bound for $\mathcal{E}$. Define

$$\bigvee \mathcal{E} = \langle \bigcup \mathcal{E} \rangle = \bigcap \{E \supset \bigcup \mathcal{E} \mid E \text{ is an equivalence relation on } X\}.$$

Then $E \subseteq \bigvee \mathcal{E}$ for all $E \in \mathcal{E}$, so $\bigvee \mathcal{E}$ is an upper bound for $\mathcal{E}$.

Proposition A.5. Let $X, \mathcal{E}(X)$ and $\mathcal{E} \subseteq \mathcal{E}(X)$ be as above. Then $\bigvee \mathcal{E}$ and $\bigwedge \mathcal{E}$ are respectively the least upper bound and the greatest lower bound of $\mathcal{E}$. Consequently, $(\mathcal{E}(X), \subseteq)$ is a complete lattice.

Proof. Suppose E_1 is a lower bound for $\mathcal{E}$. Then, by definition $E_1 \subseteq E$ for all $E \in \mathcal{E}$. But then $E_1 \subseteq \bigcap \mathcal{E}$, so $\bigwedge \mathcal{E}$ is the meet of $\mathcal{E}$.

Suppose E_0 is an upper bound for $\mathcal{E}$. Then, by definition $E \subseteq E_0$ for all $E \in \mathcal{E}$. But then $\bigcup \mathcal{E} \subseteq E_0$, and since E_0 is an equivalence relation, also $\langle \bigcup \mathcal{E} \rangle \subseteq E_0$. So $\bigvee \mathcal{E}$ is the join of $\mathcal{E}$.

B APPENDIX: PROOFS

B.1 Proof of Theorem 2.11

We will show the construction for F and G and leave the rest of the proof to the reader because it would go beyond the scope of the present paper. A reader familiar with the corresponding areas (computability, descriptive set theory) can readily verify (2) for the construction of F below.

Suppose $\mathcal{X} = (X, S \times M, T)$ is an SM-system. Let $X_s = X$ and $X_m = T$. Thus, for each transition in $\mathcal{X}$, we will have a motor state in $F(\mathcal{X})$. Define

$$T_0 = \{(x, s, t) \mid (\exists y, m)((x, (s, m), y) = t)\}$$

and

$$T_1 = \{(t, m, y) \mid (\exists x, s)((x, (s, m), y) = t)\}$$

be disjoint copies of X and define $F(\mathcal{X}) = (X_s \cup X_m, S \cup M, T_0 \cup T_1)$.

On the other hand, given $\mathcal{X} \in \mathrm{aSM}$, $\mathcal{X} = (X_s \cup X_m, S \cup M, T)$, let $G(\mathcal{X}) = (X', S \times M, T')$ where $X' = X_s$ and $(x, (s, m), y) \in T'$ if and only if there exists $z \in X_m$ such that $(x, s, z) \in T$ and $(z, m, y) \in T$.

B.2 Proof of Lemma 2.19

Again, we will only show the constructions of the functions and leave the rest of the proof to the reader. Let $\mathcal{F}$ be the class of quasifilters, $\mathcal{P}$ the class of quasipolicies, and $\mathcal{L}$ the class of labeled transition systems. We will define bijections $\mathrm{LTS}_F\colon \mathcal{F} \to \mathcal{L}$ and $\mathrm{LTS}_P\colon \mathcal{P} \to \mathcal{L}$ The constructions are dual to each other. Suppose $\mathcal{X} = (X, S \times M, T)$ is a quasifilter. Let $h\colon X \to S$ be the function defined by $h\colon x \mapsto s_x$ where s_x is as in Definition 2.12. Now let $\mathrm{LTS}_F(\mathcal{X}) = (X, M, T_M, h, S)$. We use S here as the set of labels and h as the labeling function to emphasize that the natural interpretation here is that h is a sensor mapping.

Now suppose that $\mathcal{X} = (X, M, A, h, S)$, $A \subseteq X \times M \times X$, is a labeled transition system. Let T be defined by

$$T = \{(x, (h(x), m), y) \mid (x, m, y) \in A\} \subseteq X \times (S \times M) \times X.$$

Then $\mathcal{X}' = (X, S \times M, T)$ is an SM-system which is a quasifilter and in fact $\mathcal{X}' = \mathrm{LTS}_F^{-1}(\mathcal{X})$. We have now $\mathrm{LTS}_F^{-1}(\mathrm{LTS}_F(\mathcal{X})) = \mathcal{X}$ for any quasifilter $\mathcal{X}$ and $\mathrm{LTS}_F(\mathrm{LTS}_F^{-1}(\mathcal{X}'))$ for any labeled transition system $\mathcal{X}'$. Similarly if $\mathcal{X} = (X, S \times M, T)$ is a quasipolicy, define $\mathrm{LTS}_P(\mathcal{X}) = (X, S, T_S, h, M)$ where $h(x) = m_x$ (see Definition 2.13). Analogously to the above we also have the inverse function LTS_P^{-1}. We have found the needed one-to-one correspondences.

In this proof we reversed the roles of S and M, now M being the set of labels, because they are thought of as the "outputs" of the policy.

B.3 Proof of Theorem 2.24

In this proof i always ranges over $\{0, 1\}$. Every time i appears below, we drop the phrase "for $i \in \{0, 1\}$". For part 3 note that $(x, y) \mapsto (y, x)$ is an isomorphism. For part 1, if $f_i\colon \mathcal{X}_i \to \mathcal{X}_i'$ are isomorphism, let $g\colon (x, y) \mapsto (f_0(x), f_1(y))$. Then g is an isomorphism from $\mathcal{X}_0 * \mathcal{X}_1$ to $\mathcal{X}_0' * \mathcal{X}_1'$. We leave the verification of these statements to the reader and proceed to prove part 2. Let R_i be the bisimulation witnessing $\mathcal{X}_i \sim \mathcal{X}_i'$. Let

$$R_0 * R_1 = \big\{((x_0, x_1), (x_0', x_1')) \in (X_0 \times X_1) \times (X_0' \times X_1') \mid (x_i, x_i') \in R_i\big\}. \tag{S1}$$

Let us show that $R = R_0 * R_1$ witnesses that $\mathcal{X}_0 * \mathcal{X}_1 \sim \mathcal{X}_0' * \mathcal{X}_1'$. Suppose that $((x_0, x_1), (x_0', x_1')) \in R$, $u \in U_0 \cap U_1$ and $(y_0, y_1) \in X_0 \times X_1$. Suppose further that $(x_0, x_1) \xrightarrow{u} (y_0, y_1)$, meaning that

$$((x_0, x_1), u, (y_0, y_1)) \in T_0 * T_1.$$

Then by the definition of coupling we have that $(x_i, u, y_i) \in T_i$. By (S1) we now have $(x_i, x_i') \in R_i$. Since R_i is a bisimulation, there is $y_i' \in X_i'$ with $(y_i, y_i') \in R_i$ and $(x_i', u, y_i') \in T_i$. By the definition of $T_0 * T_1$ we have now $((x_0', x_1'), u, (y_0', y_1')) \in T_0 * T_1$, which completes the proof that R is a bisimulation.

B.4 Proof of Proposition 2.27

Let $R = X \times X'$. We need to show that R is a bisimulation. In the definition of bisimulation, Definition 2.2, the conclusion of the implication that needs to be satisfied is "there exists $y' \in X'$ with $(x', u, y') \in T'$ and $(y, y') \in R$". However, because both R and T' contain "everything", this is trivially true as long as $X \times X'$ is non-empty. However, if it is empty, then the premise of that

implication is false (no $(x, x') \in R$ exist); thus, the definition of bisimulation is again trivially satisfied.

B.5 Proof of Proposition 2.37

We now show that T' and f are well defined. For that note that by Definition 2.34 we have $[x]_h = [x]_{E^h} = h^{-1}(x)$, and for all $y \in [x]_h$ we have $h(y) = h(x)$. On the other hand, if $[y]_h \neq [x]_h$, then $h(y) \neq h(x)$ and so f is injective. It is surjective by the definition of S'. Finally, $([x]_h, u, [y]_h) \in T/h$ if and only if $(h^{-1}(h(x)), u, h^{-1}(h(y))) \in T/h$ if and only if $(h(x), u, h(y)) \in T'$ (by the definition of T') if and only if $(f([x]_h), u, f([y]_h)) \in T'$ (by the definition of f).

B.6 Proof of Proposition 4.4

Suppose $E \subseteq X$ is a bisimulation and let $x_1, x_2 \in X$ be such that $(x_1, x_2) \in E$ and let $u \in U$. Let $x_1' = \tau(x_1, u)$. Since E is a bisimulation, there exists x_2' such that $\tau(x_2, u) = x_2'$ and $(x_1', x_2') \in E$. But $\tau(x_2, u)$ is uniquely determined and so it follows that $(\tau(x_1', u), \tau(x_2', u)) \in E$. Suppose now that E is a sufficient equivalence relation on X. We will show that it is a bisimulation. Since E is symmetric, $E^T = E$, so it is sufficient to show that E is a simulation. Suppose $(x_1, x_1') \in E$ and and $u \in U$. By the assumption that (X, U, τ) is an automaton and E is sufficient, we have that $(x_2, x_2') \in E$ where x_2 and x_2' are the unique elements such that $\tau(x_1, u) = x_2$ and $\tau(x_1', u) = x_2'$. This proves that E is a simulation as needed.

B.7 Proof of Proposition 4.5

Suppose $\mathcal{X}/h$ is an automaton. That means that T/h is a function with domain $X/h \times U$, so $\mathcal{X}/h$ must be full. For sufficiency assume that $x_1, x_2 \in X$ and $u \in U$ are such that $h(x_1) = h(x_2)$, so in particular $[x_1]_h = [x_2]_h$. Suppose further that x_1', x_2' are such that $(x_1, u, x_1'), (x_2, u, x_2') \in T$. We need to show that then $h(x_1') = h(x_2')$. We have $([x_1]_h, u, [x_1']_h) \in T/h$ and $([x_2]_h, u, [x_2']_h) \in T/h$. Since T/h is a function, and $[x_1]_h = [x_2]_h$, we must have $[x_1']_h = [x_2']_h$ which by definition means that $h(x_1') = h(x_2')$, so h is sufficient. For the other direction, suppose that h is sufficient and that $\mathcal{X}$ is is full. We want to show that for all $[x_1]_h \in X/h$ and $u \in U$ there is a unique $[x_2]_h \in X/h$ with $([x_1]_h, u, [x_2]_h) \in T/h$. Existence follows from fullness. For uniqueness and assume that

$$([x_1]_h, u, [x_2]_h), ([x_1]_h, u, [x_2']_h) \in T/h \tag{S2}$$

for some $x_1, x_2, x_2' \in X$. Since they are chosen to arbitrarily, it is enough to show that $h(x_2) = h(x_2')$, because this implies that $[x_2]_h = [x_2']_h$ and so $[x]_h$ together with u uniquely determine the element $\xi = [x_2]_h = [x_2']_h$ such that $([x_1]_h, u, \xi) \in T/h$. By (S2) there must be $z_1, z_1' \in [x_1]_h$ and $z_2 \in [x_2]_h$, $z_2' \in [x_2']_h$ such that $(z_1, u, z_2), (z_1', u, z_2') \in T$. Since $h(z_1) = h(z_1')$ and h is sufficient, we have $h(z_2) = h(z_2')$ and since $h(z_2) = h(x_2)$ and $h(z_2') = h(x_2')$ we have that $h(x_2) = h(x_2')$ as needed.

B.8 Proof of Proposition 4.8

The intuition is that if we can predict the future from "nothing", it means that the future is always the same. Let us prove the first statement. The second statement then follows because E^h has exactly one equivalence class if and only if h is constant. Plugging in $n = 0$ into Definition 4.7, it says "...if there are no two $(T, E, 0)$-equivalent 0-chains $(x_0), (x_0')$ with $(x_0, x_0') \notin E$". However, the (T, E, n)-equivalence only concerns elements of the sequence before the nth element, before 0th in this case, which do not exist. Thus, all 0-chains are equivalent by definition. Hence, the definition

of 0-sufficiency becomes "...if there are no two sequences $(x_0), (x'_0)$ with $(x_0, x'_0) \notin E$". This amounts to saying that E has one equivalence class.

B.9 Proof of Theorem 4.9

1-sufficiency says that for all 1-chains (x_0, u_0, x_1) and (x'_0, u_1, x'_1), if x_0 and x'_0 are equivalent and $u_0 = u_1$, then x'_0 and x'_1 are equivalent which is exactly the definition of sufficiency.

B.10 Proof of Theorem 4.10

The intuition here is that "knowing more doesn't hurt". We prove the statement for equivalence relations. Suppose E is n-sufficient and $m > n$. Suppose to the contrary that E is not m-sufficient. This is witnessed by some m-chains

$$c = (x_0, u_0, \ldots, x_{m-1}, u_{m-1}, x_m) \text{ and } c' = (x'_0, u'_0, \ldots, x'_{m-1}, u'_{m-1}, x'_m)$$

which are (T, E, m)-equivalent and $(x_m, x'_m) \notin E$. Now define restrictions c_0 and c'_0 which are obtained from c and c' by ignoring all elements with indices less than $(m - n)$. Then c, c' are n-chains and are in fact (T, E, n)-equivalent. The last elements are still x_m and x'_m and $(x_m, x'_m) \notin E$, which means that c_0, c'_0 witness that E is not n-sufficient and contradicts our assumption.

B.11 Proof of Theorem 4.15

For $\wedge$: Suppose $x, x' \in X$ are such that $(x, x') \in \bigwedge \mathcal{E}$ and $u \in U$. Since $\bigwedge \mathcal{E} = \bigcap \mathcal{E}$, we have that $(x, x') \in E$ for all $E \in \mathcal{E}$. Since all $E \in \mathcal{E}$ are sufficient, it follows that $(\tau(x, u), \tau(x', u)) \in E$ for all $E \in \mathcal{E}$, and so $(\tau(x, u), \tau(x', u)) \in \bigcap \mathcal{E} = \bigwedge \mathcal{E}$.

For $\vee$: Suppose $x, x' \in X$ are such that $(x, x') \in \bigvee \mathcal{E}$ and $u \in U$. By the definition of $\bigvee \mathcal{E}$ there exist $z_1, \ldots, z_k \in X$ such that $x = z_1$, $x' = z_k$ and for all $i < k$ there is $E_i \in \mathcal{E}$ such that $(z_i, z_{i+1}) \in E_i$. By the sufficiency of each E_i, we have then that $(\tau(z_i, u), \tau(z_{i+1}, u)) \in E_i$ and so the sequence $\tau(z_1, u), \ldots, \tau(z_k, u)$ witnesses that $(\tau(x, u), \tau(x', u)) \in \bigvee E$.

B.12 Proof of Theorem 4.19

Let $\mathcal{E}$ be the set of all sufficient equivalence relations $E \subseteq E_0$. Then $\mathcal{E}$ is non-empty, because the identity-relation $\{(x_1, x_2) \in X^2 \mid x_1 = x_2\}$ is a sufficient refinement of E_0. Let $E = \bigvee \mathcal{E}$. By Lemma A.3 we have $E \subseteq E_0$, so E is a refinement of E_0, and by Theorem 4.15 E is sufficient. On the other hand if E' is a sufficient refinement of E_0, then $E' \in \mathcal{E}$ and so $E' \subseteq E$, so E is $<_r$-minimal. The same argument proves the uniqueness too: if E' is another minimal sufficient refinement of E_0, then again $E' \in \mathcal{E}$ and so $E' \subseteq E$. But $E' \subsetneq E$ would contradict the minimality of E', so we must have $E' = E$.

B.13 Proof of Theorem 4.23

Suppose $(x, x') \in E_f$. Then there is n such that $x = f^n(x')$. Now

$$\tau(x, u) = \tau(f^n(x'), u) = f^n(\tau(x', u)).$$

The last equality follows from the fact that f is an automorphism. By definition, this means that $(\tau(x, u), \tau(x', u)) \in E_f$.

B.14 Proof of Theorem 4.24

Since E is closed under f, E_f is a refinement of E. By Theorem 4.23, E_f is also sufficient, so by $\leqslant_r$-minimality of E', we have $E_f \subseteq E'$ which implies that E' is closed under f.

B.15 Proof of Claim 4.25

We will show that the equivalence relation E^{h_1} is the mininimal sufficient refinement of E^h (Definition 2.34). Let $f\colon X_1 \to X_1$ be defined by $f(x,b) = (-x,-b)$. Then f is an automorphism of $\mathcal{X}_1$ and $E_f = E^{h_1}$, so E^{h_1} is sufficient and any minimal sufficient refinement E of E^h must satisfy $E \leqslant_r E^h$.

In the base labeling h, moving forward from $(1,1)$ results in a different sensation than moving forward from $(1,-1)$, so they must be E-non-equivalent. But then inductively this also applies to their neighbours $(0,1)$ and $(0,-1)$ as well as $(2,1)$ and $(2,-1)$ and so on. Thus E^{h_1} is in fact minimal.
